# Phylogeny and Differentiation of Reptilian and Amphibian Ranaviruses Detected in Europe

**DOI:** 10.1371/journal.pone.0118633

**Published:** 2015-02-23

**Authors:** Anke C. Stöhr, Alberto López-Bueno, Silvia Blahak, Maria F. Caeiro, Gonçalo M. Rosa, António Pedro Alves de Matos, An Martel, Alí Alejo, Rachel E. Marschang

**Affiliations:** 1 Fachgebiet für Umwelt- und Tierhygiene, Universität Hohenheim, Stuttgart, Germany; 2 Centro de Biología Molecular Severo Ochoa (Consejo Superior de Investigaciones Científicas-Universidad Autónoma de Madrid), Madrid, Spain; 3 Chemisches und Veterinäruntersuchungsamt Ostwestfalen Lippe (CVUA-OWL), Detmold, Germany; 4 Centro de Estudos do Ambiente e do Mar (CESAM) Lisboa, Lisbon, Portugal; 5 Departamento de Biologia Vegetal, Faculdade de Ciências da Universidade de Lisboa, Lisbon, Portugal; 6 Durrell Institute of Conservation and Ecology, School of Anthropology and Conservation, University of Kent, Canterbury, United Kingdom; 7 Institute of Zoology, Zoological Society of London, Regent’s Park, London, United Kingdom; 8 Centre for Ecology, Evolution and Environmental Changes (CE3C), Faculty of Sciences, University of Lisbon, Lisbon, Portugal; 9 Centro de Investigação Interdisciplinar Egas Moniz (CiiEM), Monte de Caparica, Portugal; 10 Department of Pathology, Bacteriology and Avian Diseases, Faculty of Veterinary Medicine, Ghent University, Ghent, Belgium; 11 Centro de Investigación en Sanidad Animal, Instituto Nacional de Investigación y Tecnología Agraria y Alimentaria, Valdeolmos, Spain; 12 Laboklin GmbH & Co. KG, Laboratory for Clinical Diagnostics, Bad Kissingen, Germany; University of Liverpool, UNITED KINGDOM

## Abstract

Ranaviruses in amphibians and fish are considered emerging pathogens and several isolates have been extensively characterized in different studies. Ranaviruses have also been detected in reptiles with increasing frequency, but the role of reptilian hosts is still unclear and only limited sequence data has been provided. In this study, we characterized a number of ranaviruses detected in wild and captive animals in Europe based on sequence data from six genomic regions (major capsid protein (MCP), DNA polymerase (DNApol), ribonucleoside diphosphate reductase alpha and beta subunit-like proteins (RNR-α and -β), viral homolog of the alpha subunit of eukaryotic initiation factor 2, eIF-2α (*v*IF-2α) genes and microsatellite region). A total of ten different isolates from reptiles (tortoises, lizards, and a snake) and four ranaviruses from amphibians (anurans, urodeles) were included in the study. Furthermore, the complete genome sequences of three reptilian isolates were determined and a new PCR for rapid classification of the different variants of the genomic arrangement was developed. All ranaviruses showed slight variations on the partial nucleotide sequences from the different genomic regions (92.6–100%). Some very similar isolates could be distinguished by the size of the band from the microsatellite region. Three of the lizard isolates had a truncated *v*IF-2α gene; the other ranaviruses had full-length genes. In the phylogenetic analyses of concatenated sequences from different genes (3223 nt/10287 aa), the reptilian ranaviruses were often more closely related to amphibian ranaviruses than to each other, and most clustered together with previously detected ranaviruses from the same geographic region of origin. Comparative analyses show that among the closely related amphibian-like ranaviruses (ALRVs) described to date, three recently split and independently evolving distinct genetic groups can be distinguished. These findings underline the wide host range of ranaviruses and the emergence of pathogen pollution via animal trade of ectothermic vertebrates.

## Introduction

The family *Iridoviridae* consists of five genera which are pathogens of invertebrates (genera: *Iridovirus*, *Chloriridovirus*), fish (genera: *Lymphocystivirus*, *Megalocytivirus*), and multiple ectothermic vertebrates (genus: *Ranavirus*). Ranaviruses are large (120–180 nm), icosahedral, double-stranded DNA viruses [1] that have been shown to be emerging pathogens of fish and amphibians [2–5], and detection of these viruses in reptiles has also been increasing [6–7]. A rapidly growing number of ranavirus variants have been detected worldwide during the last years in a wide range of wild and captive hosts, but most of them have not yet been sufficiently studied. Numerous viruses have only been characterized based on a 500 bp portion of the ranaviral major capsid protein (MCP) gene. This structural protein is commonly used in diagnostics due to its highly conserved sequence, but this reduces its use in distinguishing among various virus strains [8–11]. For this reason, several studies of amphibian and piscine ranaviruses have focused on the determination of more variable genomic regions, which can be used for virus differentiation (e.g. [12–14]). Some of the established PCRs were designed to obtain the complete MCP gene sequence, others targeted genes involved in virus replication (e.g. DNA replication and repair, transcription of DNA, nucleotide metabolism). Due to new sequencing technologies, an increasing number of ranaviruses isolated from amphibians (*Frog virus 3* (FV3) [15], tiger frog virus (TFV) [16], *Ambystoma tigrinum virus* (ATV) [17], common midwife toad virus (CMTV) [18], Rana grylio virus (RGV) [19], Andrias davidianus ranavirus (ADRV) [20, 21]), fish (Singapore grouper iridovirus (SGIV) [22], grouper iridovirus (GIV) [23], *Epizootic haematopoietic necrosis virus* (EHNV) [24], European sheatfish virus (ESV) [25]), and one single reptilian ranavirus (soft-shelled turtle iridovirus (STIV) [26]) have also been completely sequenced. These analyses have provided preliminary information about the evolutionary history of these emerging viruses including undergone host shifts between different vertebrate classes. Ranaviruses are currently subdivided into the amphibian-like ranaviruses (ALRV) and the grouper iridovirus (GIV)-like ranaviruses, which have only been found in fish so far [24]. To date, full-length genome sequences from ALRV have been published from isolates detected in Asia (TFV, STIV, RGV, ADRV), America (FV3, ATV), Australia (EHNV), and Europe (CMTV, ESV). It has been demonstrated that these viruses can be divided into three groups based on their different genomic structures [18, 24]. A total of 98 putative open reading frames (ORFs) were identified in these full-length ranaviral genomes. The specific role of most ORFs is still unclear [15], but it has been speculated that several ORFs conserved among ranaviruses play important roles in virulence by acting as immune evasion or host range genes [27]. Recent studies proposed a quick differentiation of individual ranaviruses (FV3/STIV and CMTV) based on the variable number of tandem repeats in the microsatellite region [18].

During the last years, an increasing number of ranaviruses have been detected in wild, captive, and imported reptiles and amphibians in Europe, which have been only partially characterized [7, 28–34]. Some of the infected animals did not show any clinical signs, whereas fatal mass-mortality events occurred in other affected animal groups. In this study, we further characterized these and other unpublished ranaviruses from a wide range of hosts based on multiple genomic regions, including large portions of MCP gene, various genes involved in virus replication (DNA polymerase (DNApol), ribonucleoside diphosphate reductase alpha and beta subunit-like protein (RNR-α and-β)), one putative virulence factor (viral homolog of the alpha subunit of eukaryotic initiation factor 2, eIF-2α (*v*IF-2α)), and the likely non-coding microsatellite region—and compared them with previously studied isolates. Moreover, we developed a PCR assay to determine the genomic arrangements of these ranaviruses and analyzed the complete genomes of three reptilian isolates. Our aims were: 1. to understand the relationships among viruses from various taxonomical groups; 2. to use that data to help elucidate the role of reptilian hosts in the epidemiology of ranaviruses; 3. to check the correlation of a putative virulence factor with documented differences in the pathogenicity of virus variants; 4. to identify suitable genomic targets for rapid ranavirus differentiation and classification; and 5. to shed light on the epidemiology of ranaviral disease in Europe.

## Materials and Methods

### Samples

A total of 18 ranaviruses from various reptiles and amphibians detected in Europe were characterized in this study and compared to previously analyzed isolates from ectothermic vertebrates. The viruses studied, their host species, the country of origin, the year of detection, and associated clinical signs are listed in Tables 1–3. Chelonian host species included Hermann’s tortoises (*Testudo hermanni*) (n = 2), an Egyptian tortoise (*T*. *kleinmanni*), and a marginated tortoise (*T*. *marginata*). Furthermore, one isolate from a snake (red blood python (*Python brongersmai*)) and six different ranaviruses from lizards were tested, namely from a leaf-tailed gecko (*Uroplatus fimbriatus*), an Iberian mountain lizard (*Iberolacerta monticola*), green striped tree dragons (*Japalura splendida*), brown anoles (*Anolis sagrei*), an Asian glass lizard (*Dopasia gracilis*), and a green anole (*Anolis carolinensis*). Seven recently detected ranaviruses from amphibians (edible frogs (*Pelophylax* kl. *esculentus*) (n = 2), Lake Urmia newts (*Neurergus crocatus*), a common midwife toad (*Alytes obstetricans*), and Bosca’s newts (*Lissotriton boscai*) (n = 3)) were also further characterized. Virus isolates were obtained from all species except the Lake Urmia newts (only DNA available).

**Table 1:** Reptilian ranaviruses included in this study.

Virus	Acronym / No.	Host species	Country of origin	Year of detection	Short case history/clinical signs	Reference(s)	GenBank IDs
***Chelonians***:							
Testudo hermanni ranavirus*	CH8/96	**Hermann’s tortoise** *Testudo hermanni*	Switzerland	1996	Stomatitis, hepatitis, liver necrosis, basophilic intracytoplasmic inclusion bodies (liver, gastrointestinal tract, lungs), bacterial coinfection. Death of all co-housed tortoises.	[28]	Complete genome: KP266741*
Tortoise ranavirus 1*	ToRV1 (882/96)	**Egyptian tortoise** *Testudo kleinmanni*	Germany	1996	Rhinitis, stomatitis, necrosis in the spleen, intracytoplasmic inclusion bodies (tongue), bacterial coinfection. 2nd animal in collection survived.	[30]	Complete genome: KP266743*
Tortoise ranavirus 2*	ToRV2 (5187/07)	**Hermann’s tortoise** *Testudo hermanni*	Germany	2007	Stomatitis, emaciation, enteritis, bacterial coinfection.	[30]	MCP: KM516713*; DNApol: KM516722*; RNR-α: KM516731*; RNR-β: KM516740*; vIF-2α: KM516751*
	ToRV2 (CU60/09)	**Marginated tortoise** *Testudo marginata*	Germany	2009	Stomatitis, necrosis in the trachea and liver, hepatitis, splenitis, pancreatitis, dermatitis and myositis in foreleg, intracytoplasmic inclusion bodies (lungs), bacterial coinfection. High mortality in mixed collection of tortoises.	[30]	
Soft-shelled turtle iridovirus	STIV	**Chinese soft-shelled turtle** *Trionyx sinensis*	China	1997	“Red neck disease”, petechial haemorrhages in the liver, high mortality in farmed animals.	[59]	EU627010
***Lizards***:							
German gecko ranavirus*	GGRV (2000/99)	**Leaf-tailed gecko** *Uroplatus fimbriatus*	Germany	2001	Granulomatous lesions on the tongue, hepatitis, only one animal in a mixed collection (other lizards + toads) died.	[29]	Complete genome: KP266742*
Lacerta monticolaranavirus*	LMRV	**Iberian mountain lizard** *Iberolacerta monticola*	Portugal (Serra da Estrela)	2003/2004	Wild-caught animal, no clinical signs reported, coinfected with erythrocytic necrosis virus.	[31]	MCP: KM516719*; DNApol: KM516728*; RNR-α: KM516737*; RNR-β: KM516746*; vIF-2α: KM516757*
Japalura splendida ranavirus*	JSpRV	**Green striped tree dragon** *Japalura splendida*	Germany (imported from Asia via Florida)	2011	Skin lesions, systemic haemorrhages, liver necrosis, large number of animals died. AdV / IIV in the same group	[32]	MCP: KM516721*; DNApol: KM516730*; RNR-α: KM516739*; RNR-β: KM516748*; vIF-2α: KM516759*
Anolis sagrei ranavirus*	ASRV	**Brown anole** *Anolis sagrei*	Germany (imported from Florida)	2008/2011	RV found repeatedly in different imported groups during 3 years. Low to high mortality, apathy, skin lesions. Coinfection with reovirus in one animal.	[7]	MCP: KM516716*; DNApol: KM516725*; RNR-α: KM516734*; RNR-β: KM516743*; vIF-2α: KM516754*
Dopasia gracilis ranavirus*	DGRV	**Asian glass lizard** *Dopasia gracilis*	Germany (imported from Asia)	2012	Illegally imported animals confiscated and divided up to different zoological organizations, a number of animals died. Skin lesions. IIV in the same animal.	[7]	MCP: KM516714*; DNApol: KM516723*; RNR-α: KM516732*; RNR-β: KM516741*; vIF-2α: KM516752*
Anolis carolinensis ranavirus*	ACRV	**Green anole** *Anolis carolinensis*	Germany (imported from Florida)	2012	Several animals in poor body condition separated, high mortality, skin lesions. AdV and IIV in the same animal.	[7]	MCP: KM516720*; DNApol: KM516729*; RNR-α: KM516738*; RNR-β: KM516747*; vIF-2α: KM516758*
*Snake*:							
Blood python ranavirus*	BPRV	**Red blood python** *Python brongersmai*	Germany (imported from Indonesia)	2007	100 animals imported, 30% developed severe diphteroid stomatitis and hepatitis. An unknown number of snakes died.	Blahak, unpublished	MCP: KM516715*; DNApol: KM516724*; RNR-α: KM516733*; RNR-β: KM516742*; vIF-2α: KM516753*

The different viruses are presented with reference to host species, country and year of first detection, short case history and references.

Virus / GenBank accession numbers highlighted bold*: new sequences were obtained during this study; sequences from the nonmarked virus were obtained from GenBank; AdV: adenovirus; IIV: invertebrate iridovirus; RV: ranavirus

**Table 2:** Amphibian ranaviruses included in this study.

Virus	Acronym / No.	Host species	Country of origin	Year of detection	Short case history/clinical signs	Reference(s)	GenBank IDs
Zuerich Pelophylax collection ranavirus 1*	ZPRV1	**Edible frog** *Pelophylax* kl. *esculentus*	Switzerland (imported from Germany)	2008	Reddening of the skin (legs, abdomen), haemorrhages in the gastrointestinal tract, mass mortality event.	[34]	KC440841; KC440846; KC440843; KC440845; vIF-2α: KM516749*
Zuerich Pelophylax collection ranavirus 2*	ZPRV2	**Edible frog** *Pelophylax* kl. *esculentus*	Switzerland (imported from unknown European country)	2010	Reddening of the skin (legs, abdomen), haemorrhages in the gastrointestinal tract, mass mortality event.	[34]	KC440842; KC440847; KC440844; KC440845; vIF-2α: KM516750*
Neurergus crocatus ranavirus*	NCRV	**Lake Urmia newt** *Neurergus crocatus*	Germany (imported from Iraq)	2011	Ulcerative dermatitis, systemic haemorrhages, high mortality.	[33]	MCP: KM516717*; DNApol: KM516726*; RNR-α: KM516735*; RNR-β: KM516744*; vIF-2α: KM516755*
Portuguese newt and toad ranavirus*	PNTRV	**Common midwife toad** *Alytes obstetricians*; **Bosca’s newt** *Lissotriton boscai*	Portugal (Serra da Estrela)	2013	unpublished	Rosa et al., unpublished	MCP: KM516718*; DNApol: KM516727*; RNR-α: KM516736*; RNBR-β: KM516745*; vIF-2α: KM516756*
*Frog virus 3*	FV3	**Leopard frog** *Lithobates pipiens*	America	1965	Renal adenocarcinoma. Type species of the genus *Ranavirus*.	[60]	AY548484
*Bohle iridovirus*	BIV	**Burrowing frog** *Lymnodynastes ornatus*	Australia	1992	Moribund tadpoles	[61]	AY187046; FJ374280; GU391286; GU391264; EF408913
Rana grylio iridovirus	RGV	**Pig frog** *Rana grylio*	China	1995	Mass mortality in cultured frogs	[62]	JQ654586
*Ambystoma tigrinum virus*	ATV	**Tiger salamander** *Ambystoma tigrinum stebbinsi*	USA	1996	Haemorrhages of the skin and internal organs, lethargy, high mortality.	[63]	AY150217
Tiger frog virus	TFV	**Tiger frog** *Rana tigrina rugulosa*	China	2000	Abdominal distension, ataxia, petechial haemorrhages in different organs, high mortality in cultured animals.	[64]	AF389451
Rana esculenta virus Italy 282/I02	REV 282/I02	**Edible frog** *Pelophylax esculentus*	Italy	unknown	Moribund tadpoles of wild frogs, diseased short after removal from their habitat.	[13]	FJ358611; FJ374275; GU391293; GU391271
Common midwife toad virus	CMTV	**Common midwife toad** *Alytes obstetricians*; **Alpine newt** *Ichthyosaura alpestris cyreni*	Spain	2007	Mass-mortality event in wild animals	[65, 66]	JQ231222
		**Water frog** *Pelophylax* spp.; **Common newt** *Lissotriton vulgaris*	Netherlands	2010	Mass-mortality event in wild animals.	[67]	
Andrias davidianus ranavirus	ADRV	**Chinese giant salamander** *Andrias davidianus*	China	2011	Epidemic disease with high mortality, systemic haemorrhage and swelling syndrome	[68]	KC865735

The different viruses are presented with reference to host species, country and year of first detection, short case history and references.

Virus / GenBank accession numbers highlighted bold*: new sequences were obtained during this study; sequences from nonmarked viruses were obtained from GenBank.

**Table 3:** Previously characterized fish ranaviruses included in this study.

Virus	Acronym	Host species	Country of origin	Year of detection	Short case history/clinical signs	Reference(s)	GenBank IDs
*Epizootic haematopoietic necrosis virus*	EHNV	**Redfin perch** *Perca fluviatilis*; **Rainbow trout** *Oncorhyncus mykiss*	Australia	1986	Haemorrhages and necroses in several tissues. Mass mortality event.	[69, 70]	FJ433873; FJ374274; GU391289; GU391267; FJ433873
*European catfish virus*	ECV	**European catfish** *Ameiurus melas*	France, Italy	1990	Haemorrhages, oedema, high mortality.	[71, 72]	FJ358608; FJ374277; GU391288; GU391266
European sheatfish virus	ESV	**European sheatfish** *Silurus glanis*	Germany	unknown	Commercial aquaculture, sudden high mortality. Haemorrhages and necroses in liver, kidneys, pancreas, gastrointestinal tract, spleen in experimental studies.	[73]	FJ358609; FJ374278; GU391290; GU391268; JQ724856
Pike-perch iridovirus	PPIV	**Pike-perch** *Stizostedion lucioperca*	Finnland	1998	No clinical signs. Causes experimentally disease in fish species.	[74]	FJ358610; FJ374276; GU391292; GU391269
Short-finned eel ranavirus	SERV	**Short-finned eel** *Anguilla australis*	Italy (imported from New Zealand)	unknown	No clinical signs. Causes experimentally disease in fish species.	[75]	FJ358612; FJ374279; GU391294; GU391272
Cod ranavirus	CodV	**Cod** *Gadus morhua*	Denmark	unknown	Ulcus syndrome in free-living populations.	[76]	GU391284; GU391282; GU391287; GU391265
Ranavirus maxima	Rmax	**Turbot** *Psetta maxima*	Denmark	unknown	No clinical signs.	[14]	GU391285; GU391283; GU391291; GU391270

The different viruses are presented with reference to host species, country and year of first detection, short case history and references.

### Virus propagation

Each virus isolate was propagated on host appropriate cell lines: the chelonian isolates grew on *Terrapene* heart cells (TH-1, ATCC: CCL-50), the snake virus on viper heart cells (VH2, ATCC: CCL-140), and the lizard and the amphibian isolates on iguana heart cell monolayers (IgH-2, ATCC: CCL-108). Viruses were isolated as described previously [34] and stored at -80°C.

### Virus purification

In case of weak PCR bands due to low amount of viral DNA and for complete sequencing of isolates, individual viruses were propagated in 175 cm^2^ tissue culture flasks (Cellstar, Greiner Bio-One GmbH) in their respective cell lines to obtain 100 mL of viral suspension. When 100% CPE was observed, the flasks underwent three rounds of freeze-thawing at -80°C. Afterwards, the suspension was centrifuged at low speed (4000xg) to remove the cell debris. The virus supernatant was then centrifuged at 30,000xg for 3 hours at 4°C. The obtained pellet was resuspended in 2 mL PBS buffer, aliquoted and stored at -80°C.

### Polymerase chain reaction

DNA was extracted from the cell culture supernatant (or the concentrated virus suspension) using the DNeasy Blood & Tissue Kit (Qiagen GmbH, Hilden, Germany). Prurified DNA was eluted in 100 μl Buffer AE. Three different PCRs targeting the major part (1402 nt) of the MCP gene in overlapping fragments, as well as previously described PCRs targeting partial sequences of the DNApol (519 nt), RNR-α (764 nt), and RNR-β (608 nt) genes were performed [8, 12–14, 28].

A PCR targeting the *v*IF-2α gene was developed using a previously published reverse primer [35] and a forward primer designed by V.G. Chinchar (personal communication). PCR reaction mixtures contained: 4 μM of each primer, 400 μM of each nucleotide (dATP, dTTP, dGTP, dCTP) (MWG Biotech AG, Ebersberg, Germany), 1x PCR buffer (670 mM Tris/HCL (pH 8.8), 160 mM (NH4)2SO4), 1.5 mM MgCl_2,_ and 2 units of *Taq* Polymerase (Taq Polymerase E, Genaxxon Bioscience GmbH, Ulm, Germany); 2 μl of viral DNA was added to 23 μl PCR mixture and cycled under the following conditions: an initial denaturing step at 94°C for 5 min, followed by 30 cycles at 94°C for 1 min, 41°C for 2 min, 72°C for 4 min, and a final extension step at 72°C for 5 min. For several samples, which gave very weak bands, a modified protocol was performed using PrimeSTAR Max DNA Polymerase (Takara Bio Inc., Shiga, Japan) according to the manufacturer’s protocol. Thermocycling conditions used were: 98°C for 5 min, followed by 35 cycles at 98°C for 10 sec, 55°C for 15 sec, 72°C for 5 sec, and a final extension step at 72°C for 5 min.

**Fig 1:**
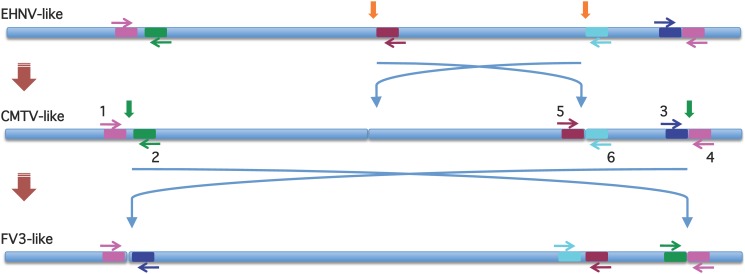
Linear schematic representation of the genomic arrangement of the three different ALRV-groups and their potential evolutionary reorganizations. Genomic inversion sites are marked by orange vertical arrows on the EHNV-like ancestor and green vertical arrows on the CMTV-like genome, and blue arrows indicate the possible inversion events. Primers targeting highly conserved sequences located around the inversion sites used to distinguish the three genomic arrangments within ALRVs are coloured arbitrarily and their position and sense indicated on all three type virus genomes.

For the visualization of the microsatellite region, a previously proposed primer pair was used [18]. The PCR reaction mixture contained 0.4 μM of each primer, 450 μM of each nucleotide, 1x PCR buffer, 1.5 mM MgCl_2_, and 1 unit of *Taq* Polymerase; 0.5 μl of viral DNA was added to 24.5 μl PCR mixture and cycled under the following conditions: an initial step at 94°C for 5 min, followed by 35 cycles at 94°C for 30 sec, 55°C for 30 sec, 72°C for 1 min, and a final extension step at 72°C for 5 min. In order to determine the genomic arrangement of the ranaviruses under study, a set of primers targeting highly conserved sequences located around the described inversion sites [18, 24] were designed based on the tortoise CH8/96 genomic sequence (Fig. 1). PCR reactions were performed using OneTaq 2x Master Mix (New England Biolabs Inc., Ipswich, MA, USA) with standard buffer following the manufacturer´s indications in a final volume of 25 μl and the following cycling conditions: one cycle at 94°C for 3 min, followed by 30 cycles at 94°C for 25 sec, 56°C for 30 sec, 68°C for 50 sec and a final extension cycle at 72°C for 5 min. The expected PCR results for the three different genomic arrangements (EHNV-like, CMTV-like, FV3-like) are presented in Table 4. Primers used in the different PCR reactions are listed in Table 5.

**Table 4:** Expected PCR results for the three different genomic arrangements.

Genome arrangement	Primer pair				
	1/2	3/4	1/3	2/4	5/6
EHNV-like	1119	411	-	-	-
CMTV-like	804	985	-	-	460
FV3-like	-	-	893	906	453

Primers are listed in Table 5; sizes of the expected PCR products for the corresponding type—viruses in bp.

**Table 5:** Primers used in PCR reactions.

Target gene	Primer	Primer position	Amplicon size (bp)	Nucleotide sequence (5’ to 3’)	Reference(s)
MCP	OL-T1	97387–97404	531	GACTTGGCCACTTATGAC	[8, 28]
	OL-T2R	97917–97899		GTCTCTGGAGAAGAAGAAT	
	MCP-BF	97813–97830	548	ACCAGCGATCTCATCAAC	[14]
	MCP-BR	98360–98341		AGCGCTGGCTCCAGGACCGT	
	MCP-5	98244–98263	585	CGCAGTCAAGGCCTTGATGT	[12]
	MCP-6R	98828–98807		AAAGACCCGTTTTGCAGCAAAC	
DNApol	DNApol-F	67188–67208	560	GTGTAYCAGTGGTTTTGCGAC	[13]
	DNApol-R	67747–67728		TCGTCTCCGGGYCTGTCTTT	
RNR-α	RNR-AF	43729–43748	806	CTGCCCATCTCKTGCTTTCT	[14]
	RNR-AR	44534–44513		CTGGCCCASCCCATKGCGCCCA	
RNR-β	RNR-BF	78029–78012	646	AGGTGTRCCRGGGYCGTA	[14]
	RNR-BR	77384–77403		GACGCTCCAYTCGACCACTT	
vIF-2α	vIF-2αF	32950–32969	247 or 1050	AAATGCAATGACTGTAAATG	[35], Chinchar, pers.comm.
	vIF-2αR	33181–33208		GGCCAAGCTTTTACACAAAGGGGCACA	
Microsatellite region	CMTVre_F	80807–80824	variable	TCTTTACTCCATCGCACA	[18]
	CMTVre_R	80913–80930		ACGCACTGAAAAGGTGCA	
Genomic arrangement	GenAr_1	14956–14980	see Table 4	GTTTGCAGAGCGTCAGCTCGTGGAC	
	GenAr_2	102673–102699		CACGAAAACTGGCAGCTGAGGGACGCC	
	GenAr_3	15849–15824		GCATGCGCAAGTCTGCCGAGGCGGTC	
	GenAr_4	103579–103551		GTGAAAGGATTGCGATAAACTGAGACCAC	
	GenAr_5	29321–29296		GACACAATCCAGCTCGTCTGTGAGAC	
	GenAr_6	28861–28889		GACTGTAGACGGCTGGCCAGGGTACGCCG	

The primer positions presented are relative to the FV3 genome (AY548484); primers of the genomic arrangement sites are relative to CH8/96 (gb KP266741).

Y = C/T, K = G/T, S = C/G, R = A/G

The obtained PCR products were separated by agarose gel electrophoresis (1.5% agarose gel (Biozym Scientific GmbH, Hessisch Oldendorf, Germany) in TAE buffer containing 0.5 μg/mL ethidium-bromide) and visualized under 320 nm UV light. PCR amplicons were gel purified using the peqGOLD Gel Extraction Kit (Peqlab Biotechnologie GmbH, Erlangen, Germany) and sent for sequencing from both directions to MWG Biotech AG (Ebersberg, Germany). The obtained PCR products from the microsatellite region were separated on a 4% agarose gel and evaluated under UV light using the Quantum ST4 imaging system (Vilber Lourmat Deutschland GmbH, Eberhardzell, Germany). The sizes of the bands were calculated with the molecular weight option. The gel bands from the PCR reactions targeting the genomic arrangement sites were evaluated manually.

### Complete sequencing: virus purification, sequencing, assembly and annotation of the viral genomes

The complete genomes from three viruses from reptiles (Hermann’s tortoise (CH8/96), Egyptian tortoise (882/96), and leaf-tailed gecko (2000/99)) were sequenced and analyzed. Viral genomes were purified as previously described [18] with some modifications. Briefly, viral particles were treated with 500 units/mL of DNAse I and S7 nucleases (Roche Diagnostics GmbH, Mannheim, Germany) to remove free DNA. After proteinase K and SDS treatment, viral DNA was extracted with phenol-chloroform and precipitated with sodium acetate and ethanol in the presence of 10 μg of glycogen from mussels (Roche Diagnostics GmbH, Mannheim, Germany) as carrier. Viral genomes were separated by electrophoresis in 0.7% agarose gels and extracted with QIAEX II gel extraction kit (Qiagen GmbH, Hilden, Germany).

For each sample, 2 μg of randomly amplified DNA (illustra GenomiPhi V2 DNA Amplification Kit; GE Healthcare, Buckinghamshire, UK) was sheared into fragments of approximately 650 bp and an indexed library constructed according to a standard protocol provided by Illumina Inc. (San Diego, CA, USA). Libraries with 800–850 bp length were pooled and sequenced with the Miseq reagent kit V2 (Illumina Inc., San Diego, CA, USA) in a Miseq sequencer hosted in the Parque Científico de Madrid. The output consisted of ca. 1.3–1.6 million of 2 x 250 bp paired-end sequences for each library. Complete genomes were assembled de novo using Newbler 2.5.3 (Roche-454 Life Science, Branford, CT, USA) under stringent parameters (97% minimum overlap identity in a 0.9 length fraction) and CLC-Genomics Workbench (trial version; 0.9 identity and 0.5 length fraction). Scaffolding was performed by overlapping contigs from both assembly technologies and gaps were filled using PCR amplification and Sanger sequencing. Specifically, sequences between positions 37436–38131 and 65674–66384 of CH8/96, 20338–21660 and 94611–95617 of 882/96 (ToRV1) and 11443–12437, 38561–39555, 51774–52773 and 80561–81341 of 2000/99 (GGRV) were confirmed. Finally, a mapping was performed with gsMapper (Newbler 2.5.3; Roche-454 Life Science, Branford, CT, USA) using a subsample of 50,000 single reads and the genomes assembled as reference to get the final genomic sequence of each virus. All three genomes had a final coverage above 5000x. Annotation was performed manually using Artemis software [36] and the similarity search algorithm BLASTP (http://www.ncbi.nih.gov/blast/) on all ORFs longer than 120 bp. Non-overlapping ORFs were numbered consecutively from the same arbitrary start point as in ATV and EHNV [24], and transcriptional sense indicated by R or L.

### Sequence analysis, phylogenetic study

Obtained partial sequences were edited, assembled and compared using STADEN Package version 2003.0 Pregap4 and Gap4 programmes [37]. The edited original sequences were compared to those in GenBank online (http://www.ncbi.nih.gov/blast/) using BLASTN. Multiple alignments of nucleotide and amino acid sequences were performed with the ClustalW algorithm of the BioEdit Sequence Alignment Editor program [38]. Sequence identity values were calculated from multiple alignments in comparison to completely sequenced ALRV (STIV, FV3, ATV, RGV, TFV, ADRV, CMTV, EHNV, ESV) and to *Bohle iridovirus* (BIV) for all sequenced genes. For the phylogenetic analysis, the gene sequences of four genes (RNR-α, RNR-β, DNApol, MCP) were concatenated (3223 bp) and aligned with corresponding gene regions of previously published ALRV sequences. Short-finned eel ranavirus (SERV) was used as an outgroup. Different phylogenetic calculations were performed in the PHYLIP program Package version 3.6. [39]—including distance based, maximum likelihood and parsimony methods—to obtain an optimal tree. Bootstrap analysis of 1000 replicates was carried out. GTR+G (general time reversible assuming gamma distribution) substitution model for MrBayes (with 1 million generations, sample frequency: 10 and burn in ratio: 25%), as well as maximum likelihood method (PhyML analysis, TIM+I+G (transition model, invariable sites, assuming gamma distribution) with 1000 bootstrap runs) were also used to reconstruct phylogenies [40] as an application of the TOPALi v2.5 program.

To compare the overall degree of nucleotide similarity of the newly sequenced complete genomes to those of other ranavirus genomes, PASC (PAirwise Sequence Comparison) software available online (http://www.ncbi.nlm.nih.gov/sutils/pasc/) was used [41]. Phylogenetic analyses of the concatenated sequences of 17 iridovirus core gene proteins (10287 aa) were performed using JTT+G (Jones-Taylor-Thornton assuming gamma distribution) substitution model for MrBayes (with 1 million generations, sample frequency: 10 and burn in ratio: 25%); maximum likelihood and neighbour-joining analyses were also carried out as applications of the TOPALi v2.5 program. Lymphocystis disease virus China (LCDV-C) was used as an outgroup.

## Results

The major part of the MCP gene and partial DNApol, RNR-α, RNR-β, and *v*IF-2α genes were successfully amplified and sequenced from all studied viruses. Two isolates detected in two distinct chelonians in two different years in Germany (a Hermann’s tortoise (isolate 5187/07) and a marginated tortoises (isolate CU 60/09)) were 100% identical to each other on all characterized genes. They were therefore considered to be the same virus and named tortoise ranavirus 2 (ToRV2). Recently obtained isolates from four different wild amphibian specimens in Serra da Estrela, Portugal (Bosca’s newts (n = 3), common midwife toad (n = 1)) were also 100% identical to each other on all sequenced genes, this virus was named Portuguese newt and toad ranavirus (PNTRV).

The sequence identities (nt and aa) of all new studied ranaviruses and completely sequenced isolates available in GenBank, as well as BIV, were calculated separately for each gene (S1–S5 Tables). The overall sequence identity of these different ranaviruses varied between the partially analyzed genes (MCP: 95.5–100%, DNApol: 97.1–100%, RNR-α: 96.4–100%, RNR-β: 97.2–100%, *v*IF-2α: 92.6–100%). Based on the partial MCP gene sequences (1332 nt), two isolates from tortoises (ToRV1 and ToRV2) and three isolates from lizards (Lacerta monticola ranavirus (LMRV), Japalura splendida ranavirus (JSpRV), and Anolis carolinensis ranavirus (ACRV)) were 100% identical to each other (S1 Table). The same three lizard ranaviruses were 100% identical to each other and to FV3 on the sequenced 764 nt of the RNR-α gene (S3 Table). Partial sequences of the DNApol genes (519 nt) of JSpRV and ACRV were also 100% identical, but differed slightly (2nt) from LMRV and FV3 (S2 Table). The partial sequence of the RNR-β gene (608 nt) from the ranavirus from another anole species (Anolis sagrei ranavirus (ASRV)) was 100% identical to the corresponding sequences in LMRV and FV3, but the other two closely related ranaviruses (JSRV, ACRV) were distinct from these and from each other on the nucleotide level (2 nt) (S4 Table).

In the PCR targeting the *v*IF-2α gene, the length of the amplified fragments clearly differed between several of the viruses. In three viruses (LMRV, JSpRV, ACRV), the size of the PCR product was approximately 250 bp and for the other eleven different ranaviruses it was approximately 1050 bp. Sequencing of the short products (211 nt) demonstrated that these sequences were 100% identical to each other and to the corresponding sequences of previously studied ranaviruses with a truncated *v*IF-2α gene (FV3, STIV). The size of the long fragments differed after aligning and cutting due to several inserts with a total length between 866 and 889 nt. ToRV1 and ToRV2 were 100% identical to each other in the analyzed partial sequences of the *v*IF-2α gene, but all other isolates were distinct from one another and from previously published ranaviruses (S5 Table). All obtained sequences of the newly studied ranaviruses have been submitted to GenBank (Tables 1–2).

**Fig 2:**
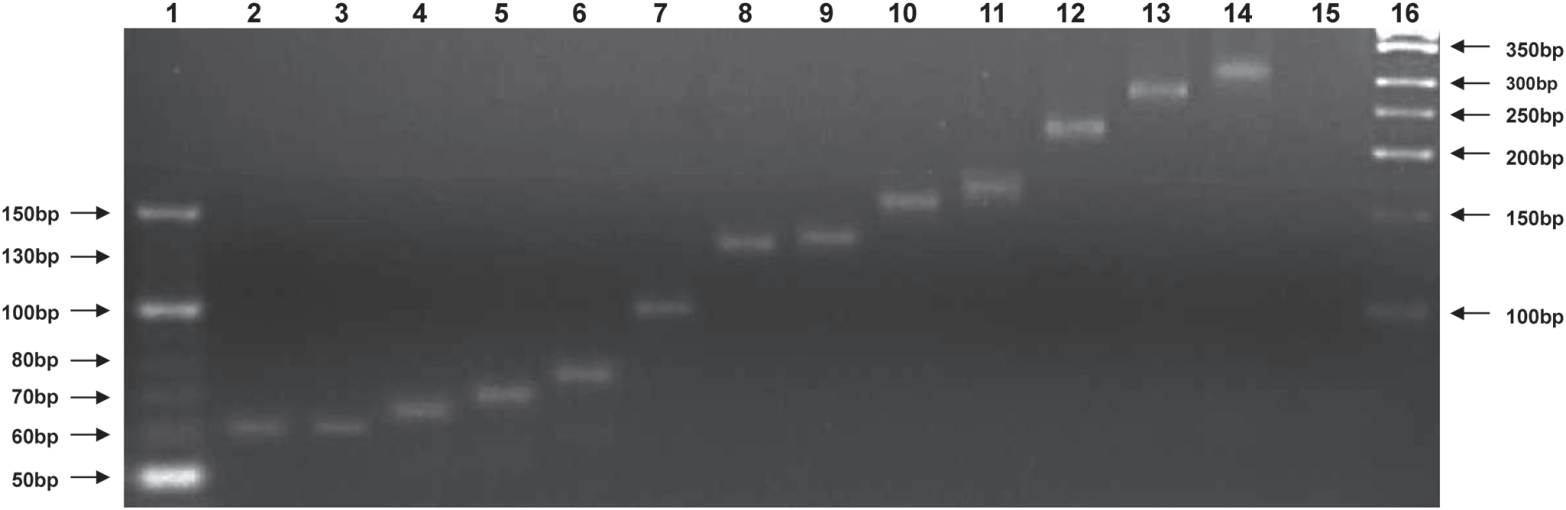
PCR amplification of the microsatellite region from the studied ranavirus isolates and FV3. The amplicons were separated by electrophoresis in 4% agarose gel. Lane 1: 10 bp marker, lane 2: ToRV1 (60 bp), lane 3: ToRV2 (62 bp), lane 4: GGRV (65 bp), lane 5: ACRV (70 bp), lane 6: ASRV (76 bp), lane7: JSpRV (101 bp), lane 8: LMRV (134 bp), lane 9: FV3 (138 bp), lane 10: DGRV (156 bp), lane 11: CH8/96 (164 bp), lane 12: ZPRV1 (230 bp), lane 13: ZPRV2 (288 bp), lane 14: BPRV (351 bp), lane 15: negative control, lane 16: 50 bp marker

The microsatellite region previously described in FV3, STIV, and CMTV was successfully amplified and visualized from all ranavirus isolates (Fig. 2, PNTRV not shown). For most isolates, the size of the amplicons clearly differed from one another (ToRV1: 60 bp, ToRV2: 62 bp, GGRV: 65 bp, ACRV: 70 bp, ASRV: 76 bp, JSpRV: 101 bp, PNTRV: 130 to 140 bp, LMRV: 134 bp, DGRV (Dopasia gracilis ranavirus): 156 bp, CH8/96 (Testudo hermanni ranavirus): 164 bp, ZPRV1 (Zuerich Pelophylax collection ranavirus 1): 230 bp, ZPRV2: 288 bp, BPRV (blood python ranavirus): 351 bp). The single virus which was not isolated in cell culture (NCRV (Neurergus crocatus ranavirus)) could not be visualized. An attempt to sequence the products failed.

The newly designed PCR for classification based on the genomic arrangements produced clear bands with the expected sizes for most viruses. CH8/96, ToRV1, ToRV2, ZPRV1, ZPRV2, and NCRV showed PCR results corresponding to CMTV-like arrangements, whereas GGRV, LMRV, JSpRV, ACRV, and DGRV were classified as FV3-like. For the remaining viruses (ASRV, BPRV, and PNTRV) no conclusive results could be obtained, possibly due to mismatches between the oligonucleotide primers and the corresponding templates.

**Fig 3:**
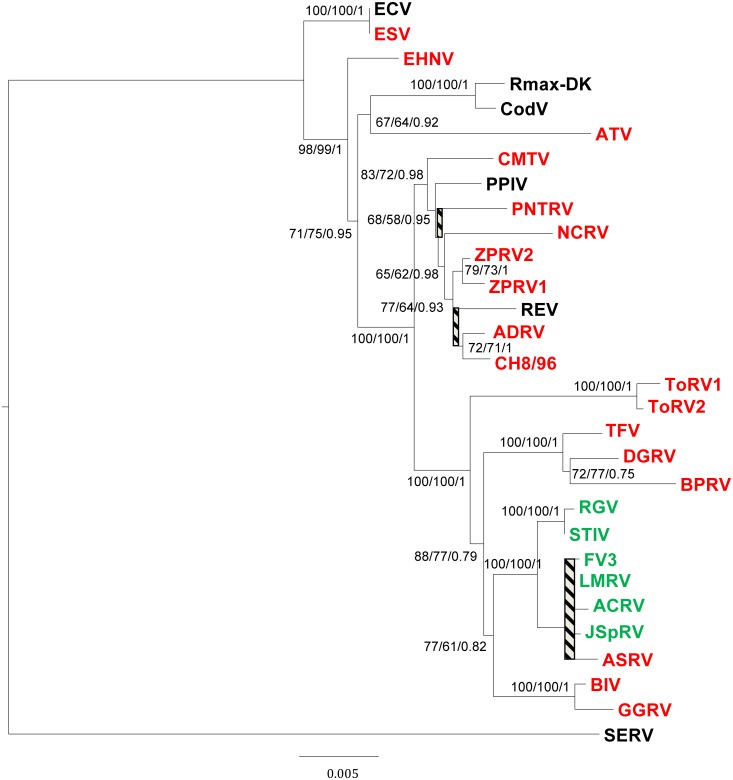
Ranavirus DNA distance tree of concatenated sequences (3223 bp) of MCP, DNApol, RNR-α and RNR-β genes. Partial nucleotide sequences of the different ranaviruses characterized in this study and ALRV sequences available in GenBank are included. Numbers at the nodes of the tree indicate bootstrap values of 1000 replicates in DNAdist-Fitch, maximum likelihood calculations, and MrBayes posterior probabilities. Branches with less than 60% support or variant clustering on the obtained trees were shaded. All calculated trees showed similar topologies. Ranaviruses with a full-length *v*IF-2α gene are indicated in red, truncated *v*IF-2α genes are in green, and those isolates for which this gene has not been sequenced are in black. GenBank accession numbers of the sequences used in the analysis: Andrias davidianus ranavirus isolate 1201 (ADRV) (KC865735), *Ambystoma tigrinum virus* (ATV) (AY150217), *Bohle iridovirus* (BIV) (AY187046, FJ374280, GU391286, GU391264), common midwife toad virus (CMTV) (JQ231222), cod ranavirus (CodV) (GU391284, GU391282, GU391287, GU391265), *European catfish virus* (ECV) (FJ358608, FJ374277, GU391288, GU391266), *Epizootic haematopoietic necrosis virus* (EHNV) (FJ433873, FJ374274, GU391289, GU391267), European sheatfish virus (ESV) (FJ358609, FJ374278, GU391290, GU391268), *Frog virus 3* (FV3) (AY548484), pike-perch iridovirus (PPIV) (FJ358610, FJ374276, GU391292, GU391269), Rana esculenta virus Italy 282/I02 (REV) (FJ358611, FJ374275, GU391293, GU391271), Rana grylio virus (RGV) (JQ654586), Ranavirus maxima (Rmax) (GU391285, GU391283, GU391291, GU391270), short-finned eel ranavirus (SERV) (FJ358612, FJ374279, GU391294, GU391272), soft-shelled turtle iridovirus (STIV) (EU627010), tiger frog virus (TFV) (AF389451), Zuerich Pelophylax collection ranavirus 1 (ZPRV1) (KC440841, KC440843, KC440845, KC440846), Zuerich Pelophylax collection ranavirus 2 (ZPRV2) (KC440842, KC440844, KC440845, KC440847).

**Fig 4:**
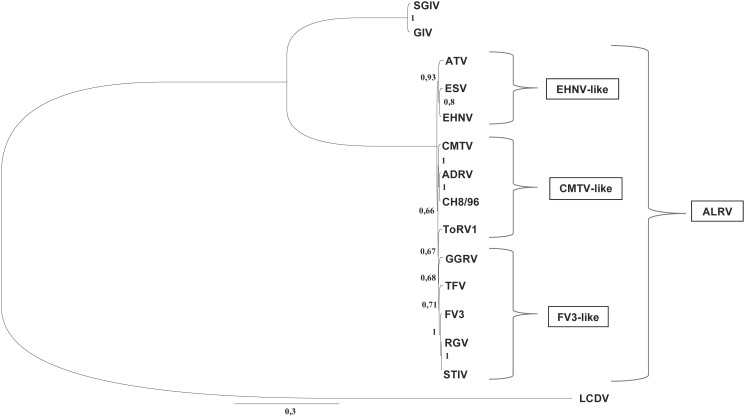
Bayesian tree of available ALRV genomes based on 17 selected core gene proteins (10287 aa). Concatenated sequences of core genes used in this analysis: Iridovirus core gene 2 (EHNV 7R)—RNApol II, a subunit; Iridovirus core gene 3 (EHNV 8L)—NTPase/ helicase; Iridovirus core gene 4 (EHNV 10L)—RAD2; Iridovirus core gene 5 (EHNV 11R)—unknown function; Iridovirus core gene 7 (EHNV 14L)—MCP; Iridovirus core gene 8 (EHNV 16L)—thiol oxidoreductase; Iridovirus core gene 9 (EHNV 18L)—deoxynucleoside kinase; Iridovirus core gene 12 (EHNV 24R)—RNAse III; Iridovirus core gene 13 (EHNV 38R)—ribonucleotide reductase, small subunit; Iridovirus core gene 14 (EHNV 43R)—RNApol II, b subunit; Iridovirus core gene 15 (EHNV 44L)—DNApol; Iridovirus core gene 17 (EHNV 53L)—myristylated membrane protein; Iridovirus core gene 19 (EHNV 72R)—unknown function; Iridovirus core gene 21 (EHNV 85L)—D5 NTPase; Iridovirus core gene 22 (EHNV 86R)—unknown function; Iridovirus core gene 23 (EHNV 89L)—serine/ threonine protein kinase; Iridovirus core gene 24 (EHNV 92L)—NTPase. Numbers at the nodes of the tree indicate MrBayes posterior probabilities of 1.000.000 replicates. Lymphocystis disease virus China (LCDV-C) was used as an outgroup. Classifications of the viruses to the different ALRV-groups based on their genomic arrangement are indicated beside the brackets. GenBank accession numbers of the sequences from ALRV used in the analysis are given in Fig. 3; EHNV(FJ433873), ESV(JQ724856), grouper iridovirus (GIV) (AY666015), Singapore grouper iridovirus (SGIV) (AY521625), LCDV-C (AY380826).

In the phylogenetic analysis of the concatenated sequences of four genes (RNR-α, RNR-β, DNApol, MCP), ACRV, ASRV, JSpRV, and LMRV clustered very closely to one another in the FV3-like clade (Fig. 3). BPRV and DGRV were closely related to TFV, and GGRV clustered most closely to BIV. ToRV1 and ToRV2 clustered very closely to one another on a separate branch, which was most closely related to the FV3/TFV/STIV group. Based on the constructed tree, CH8/96, NCRV, PNTRV, and ZPRV1/2 grouped together with CMTV/REV/PPIV and ADRV. Overall, these observed phylogenies were confirmed by the calculated Bayesian tree of the completely sequenced isolates (Fig. 4), but analyses using other phylogenetic methods (maximum likelihood, neighbour-joining) showed a slightly different clustering (data not shown). Due to lack of the corresponding gene sequences of BIV, its similarity to GGRV could not be shown. This isolate and ToRV1 clearly branched together and with FV3 in the FV3-like clade on this tree.

**Fig 5:**
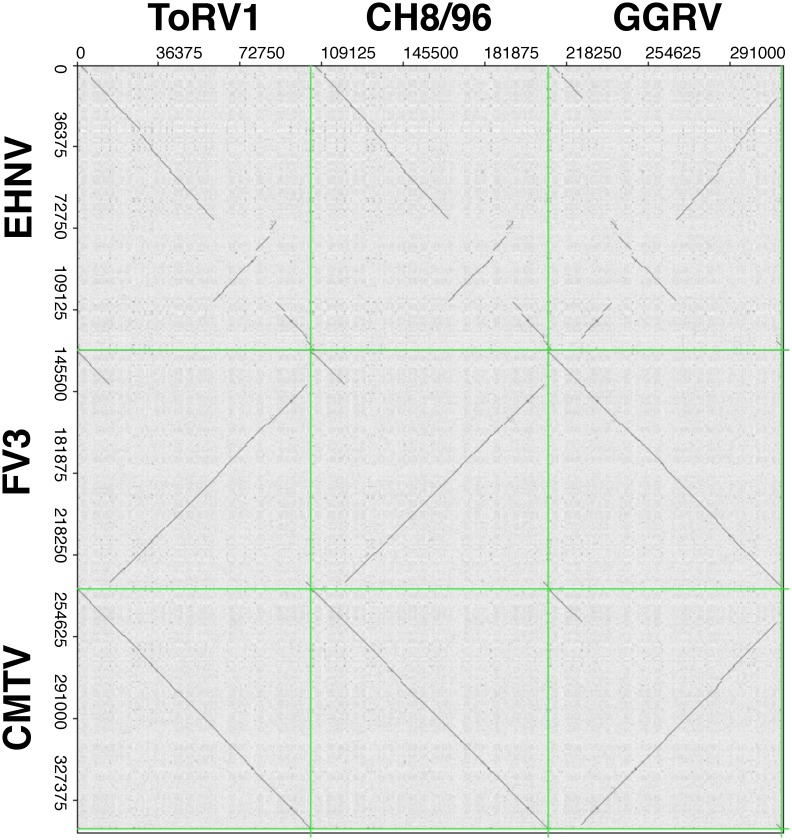
Dot plot analysis of the new sequenced isolates (ToRV1, CH8/96, and GGRV) versus other ranaviruses. Complete genomes are compared to the three described genomic arrangements in ALRV, exemplified by EHNV, FV3, and CMTV.

The complete genome sequences of three selected reptilian ranaviruses—GGRV, ToRV1, and CH8/96—were obtained (Table 1). The genomes ranged from 103681 to 105811 nucleotides in length, with an average GC content of 55%, within the range of other complete ALRV genomes. In our annotation, 73 to 76 putative ORF were identified, including orthologues for all conserved ranavirus core genes, representing an average coding capacity of 0.72 genes per kb, similar to that of EHNV (0.79 genes / kb) (S6 Table). The results of a comparison of the overall degree of nucleotide similarity of these viruses to other ranaviruses using the PASC software is presented in Table 6: when positional information was discarded (blast-based PASC), all three viruses showed identity values above 83% to ALRVs, but below 35% to the marine fish ranaviruses GIV and SGIV. The three novel reptilian ranaviruses were also shown to be distinct from each other and most similar to either CMTV or ADRV. When overall nucleotide composition (global alignment) was analyzed, lower identity values among ALRVs are obtained, reflecting the existence of three different genomic arrangments within this group [18, 24]. In this case, GGRV was found to be most similar to FV3, suggesting that these viruses are colinear. A dot plot analysis of the three genomes (Fig. 5) showed that, while GGRV is colinear with FV3, both ToRV1 and CH8/96 have the same genomic arrangment as CMTV.

**Table 6:** Analysis of ranavirus genomes using PASC (PAirwise Sequence Comparison) software.

	**BLAST-based alignments**			**global alignments**		
**Virus**	CH8/96	ToRV1	GGRV	CH8/96	ToRV1	GGRV
CH8/96		94.1	94.8		90.4	59.3
ToRV1	94.1		94.0	90.4		59.0
GGRV	94.8	94.0		59.3	58.9	
FV3	95.2	93.6	94.0	58.7	58.4	**91.5**
STIV	95.0	93.7	94.1	58.7	58.3	91.4
RGV	95.0	93.7	94.2	58.8	58.3	91.4
TFV	94.8	94.0	94.5	57.2	57.3	90.2
CMTV	96.8	**94.7**	**94.9**	94.8	**92.2**	59.2
ADRV	**97.2**	93.4	94.4	**95.1**	89.9	59.2
ATV	90.1	89.4	88.8	74.5	73.6	55.5
EHNV	86.6	85.4	85.2	71.0	70.2	46.4
ESV	85	83.5	83.5	70.3	69.5	47.3
GIV	33.9	34.2	34.3	38.7	38.6	38.7
SGIV	33.7	34.4	34.3	38.8	38.7	38.8

The complete genome sequences of the newly studied isolates (CH8/96, ToRV1, GGRV) are compared to previously sequenced ranaviruses. Results of BLAST-based alignments (do not take into account the position of DNA sequences, i.e. genomic rearrangements) and global alignments are shown in percent. The highest identity for each virus is highlighted bold. Full virus names are given in Figs. 3 and 4.

## Discussion

Ranavirus infections in amphibians, as well as EHNV infection in fish are listed as notifiable diseases by the Office International des Epizooties [42]. Unfortunately, the recommended molecular techniques for identifying ranaviruses at genus and species level using restriction endonuclease analysis (REA) developed by Marsh [43] or sequencing of a short portion of MCP gene are not commonly used in routine diagnostics any more, as these methods do not include newly described ranavirus strains and do not reflect the current state of scientific knowledge. In recent investigations of fish ranaviruses, rapid differentiation of various isolates by REA of DNApol and neurofilament triplet H1-like protein gene, as well as sequencing of different genomic regions has been proposed [13, 14]. Most of our studied isolates were distinguishable from one another on the partially sequenced genes. However, closely related strains (e.g. ToRV1 and ToRV2) were 100% identical to each other on some genes (MCP, *v*IF-2α), but showed differences on available sequences of the other genes. The slight differences between the FV3-like ranaviruses in this analysis (ACRV, ASRV, JSpRV, LMRV) could only be demonstrated on the concatenated sequences of at least three genes (MCP, DNApol, RNR-β). Analyses of the obtained complete genomic sequences of ToRV1, CH8/96, and GGRV confirmed that these isolates are typical ALRV, as expected from the previous results.

The different ranavirus isolates included in this study were obtained from animals with or without clinical signs of disease. A number of environmental and host factors, as well as different virus strains and specific combinations of host and virus genotypes seem to impact the development of disease [4, 44]. *v*IF-2α, which is only present in ALRV, appears to play an important role in the pathogenesis of ranaviruses. It has been shown experimentally that the vIF-2α from Rana catesbeiana virus Z (RCV-Z) is a functional inhibitor of human and zebrafish antiviral protein kinase R (PKR) [45]. This protein seems therefore to prevent the inactivation of eIF-2α, the inhibition of translation initiation, and the final block of viral replication. Knockout experiments with ATV confirmed that the lack of *v*IF-2α results in an increased time to death [46]. Sequencing work demonstrated that several ranaviruses (FV3, STIV) carry only a truncated version of the *v*IF-2α gene that lacks the N-terminal binding domain for the PKR and the central helical domains [15, 26]. Previous studies have provided evidence that these missing domains are required to inhibit PKR and to down-regulate the host’s innate immune response, leading to the hypothesis that these deletions might result in attenuated viruses [45, 47]. Although a ranavirus with a full-length *v*IF-2α gene (RCV-Z) was experimentally more pathogenic than FV3 [47], recent experiments demonstrated that the truncated *v*IF-2α gene also contributes to virulence [27]. In our study, one ranavirus with a truncated *v*IF-2α gene (LMRV) was isolated from an animal which did not show any clinical signs, but two other isolates (JSpRV, ACRV) from lizards which also did not have a full-length *v*IF-2α gene were detected in groups of animals with high mortality rates (Table 1). These findings strengthen the hypothesis that a second protein may also play a role in blocking PKR activity [27, 45].

It is known that anthropogenic stressors are increasing the emergence of ranavirus infection (reviewed in [4, 48]). Experimentally induced inflammation in amphibians (*Xenopus laevis*) has been shown to reactivate quiescent FV3 infection resulting in high mortality rates [49]. It is therefore possible, that the coinfections with other pathogens, including parasites and viruses (adenovirus/ invertebrate iridovirus) detected in these animals, as well as the likely general immunosuppressed state of these newly imported animals may have contributed to the clinical outcome of disease. With improving diagnostic methods, multiple viral infections are being increasingly reported in reptiles [7, 50]. The role of coinfections with various viruses is not yet understood, however, in the case of imported animals and pathogen pollution, it is to be expected that animals may have been exposed to multiple infectious agents and these may work together to determine clinical course of disease as well as immune response, length of infection and level of shedding.

All other sequenced ranaviruses had a complete *v*IF-2α gene. It is worth noting that the partial *v*IF-2α gene sequences obtained from most viruses with a full-length *v*IF-2α gene differed from one another (except ToRV1/2), whereas all truncated genes were 100% identical in the studied gene sequences. Investigations of these viruses under laboratory conditions could help to assess their individual pathogenic potential.

In previous analyses of completely sequenced ranavirus genomes, a microsatellite consisting of tandemly repeated CA dinucleotides has been found in FV3 (34 repeats, 130 bp), STIV (34 repeats, 130 bp), and CMTV (60 repeats, 180 bp) [15, 18, 26]. Although the biological function of this region is still unclear, it has been proposed to use this unique region, which does not exist in other iridoviruses, for differentiation of ranaviruses. The PCR amplification of the microsatellite region was successful in all ranavirus isolates and most of the visualized bands differed clearly from one another, although the size of the amplified fragment from FV3 (138 bp) varied slightly from the number of repeats previously detected by full genome sequencing (Fig. 2). This may result from the comparatively imprecise method or can be caused by mutation during virus propagation. Interestingly, the closely related FV3-like isolates (ACRV, ASRV, JSpRV, LMRV) could be differentiated precisely from one another and from FV3. On the other hand, the amplicons obtained from two isolates from tortoises (ToRV1 and ToRV2) were almost identical to one another. The size of the bands from the isolates from the different amphibians from Portugal, which were considered to be the same virus based on the available sequences, differed from 130 bp (Bosca’s newts (n = 2)) to 140 bp (Bosca’s newt (n = 1) and common midwife toad (n = 1)). It is therefore possible that the animals were infected with slightly diverse virus strains, which could not be differentiated based on the partially sequenced genes used in this study. Even though this PCR was not successful for non-isolated ranaviruses, it is a new tool for quick differentiation of variable ranavirus isolates.

Spread of ranavirus infection within a mixed collection of Mediterranean tortoises (marginated tortoises, Hermann’s tortoises, and spur-thighed tortoises (*T*. *graeca*)) resulting in high mortality in this group of animals, was documented for one of the studied isolates [30]. Earlier experimental and phylogenetic studies on ranaviruses have demonstrated that some isolates may not only be able to be transmitted between animal families, but even between different classes of ectothermic vertebrates. For example an amphibian ranavirus (BIV)—originally isolated from a diseased ornate burrowing frog—has been shown experimentally to be pathogenic to other species of frog, as well as to fish species and to hatchling tortoises [51, 52]. It has been shown previously that the phylogenetic analyses of ranaviruses based on concatenated sequences of 26 core genes are consistent with other genomic analyses and can be used to infer host switching [24, 53]. The phylogeny of the constructed tree based on the concatenated sequences obtained from four genes (Fig. 3) was confirmed in the Bayesian tree obtained using 17 core genes from the completely sequenced isolates (Fig. 4). The slight variations between trees obtained by different phylogenetic methods based on ALRV full-length genomes may reflect the relatively low probability due to limited available sequence data. Remarkably, one of our studied lizard ranaviruses (GGRV) clustered most closely to BIV in the phylogenetic study and most of the other characterized reptilian ranaviruses were also more closely related to amphibian ranaviruses than to viruses originally detected in reptiles. Studies on the poorly understood evolutionary history of ranaviruses suggest that the ancestral ranavirus was a fish virus and that several host shifts (from fish to frogs, from fish to salamanders, and from frogs to reptiles) have taken place. Based on the genomic structure, phylogenies and gene content of full-length ALRV genomes, it has been proposed to distinguish the probable evolutionarily oldest group including EHNV and ATV from the younger FV3/TFV/STIV group [24]. The first completely sequenced European ranavirus (CMTV) seems to occupy an intermediate position within these two lineages, which also correlates with its virulence to different amphibian orders [18]. More recently, the complete genome sequence of ADRV isolated in China from giant salamanders was also shown to be fully colinear with CMTV [20, 21]. Supporting this theory, our newly studied amphibian ranaviruses detected in anuran (ZPRV1 and ZPRV2), urodele (NCRV), and mixed host species (PNTRV) all group on the CMTV-like branch. Analyses of the genomic arrangement via the newly developed PCR assay confirmed this classification, and dot plot analyses (Fig. 5) demonstrated that ToRV1 and CH8/96 are colinear with CMTV, whereas GGRV has the same genomic arrangement as FV3. The fact that some of our reptilian isolates were related to the FV3-like group, whereas others clustered more closely to the CMTV-like lineage, strengthen the theory that a host jump from frogs to reptiles took place recently, but does however contradict the speculation that the FV3-like viruses may have produced reptile-specific viruses [18]. On checking the phylogeny based on the lengths of the vIF-2α genes, all ranaviruses with truncated genes branched very close to one another in the FV3-like group (Fig. 3) supporting previous findings on sequence gain and loss during ranaviral evolution [53]. Recent experiments proved that an FV3-like ranavirus that was isolated from a pallid sturgeon (*Scaphirhynchus albus*) during a mass mortality event [54] can be transmitted among frogs, fish, and turtles via previously exposed animals through water and that subclinically infected fish and reptiles might serve as reservoirs [55]. The presented phylogenies, as well as the analyses of the genomic arrangements show that probably not only ranaviruses from the FV3-like group, but also CMTV-like viruses have the capacity to infect amphibians, reptiles, and fish. However, the complex mechanisms leading to development of disease in supposedly low susceptible species have not been sufficiently studied.

Phylogeographic studies have been shown to be a valuable method for understanding the origin and mechanisms of ranavirus spread [56]. It is interesting to note that ranaviruses in Europe differ significantly from one another and that the phylogenetic similarity of the newly studied viruses does not correlate with the relationships of their host species, but clearly reflects their geographic origin: most of the FV3-like isolates were obtained from animals which had been imported from or via the USA (ACRV, ASRV, JSpRV), whereas the isolates detected in snake/lizard from Asia (BPRV, DGRV) cluster most closely to a Chinese ranavirus (TFV), and the European ranaviruses (PNTRV, ZPRV1/2, CH8/96), as well as the ranavirus from Iraq (NCRV) form a separate group with other ranaviruses detected in Europe (CMTV, PPIV, REV) (Fig. 3). These findings indicate that some of the detected ranaviruses represent original European strains, whereas most isolates seem to be introduced from other geographic regions. However, some isolates did not cluster with other isolates from their geographic origin (LMRV, GGRV, ADRV) and the phylogenetic position of two viruses could not clearly be determined (ToRV1 and ToRV2). During studies on the emergence of ranavirus infection in wild amphibians in northern Spain, a number of highly virulent CMTV-like, as well as one FV3-like virus have been found [57]. The origins of this low virulent virus, as well as of the Portuguese FV3-like isolate included in our study (LMRV) are unclear, but it remains possible that FV3-like viruses had been introduced to the Iberian Peninsula, but have not caused disease in the native animal populations. The fact that ToRV1 branches most closely to the FV3-like group (Fig. 4), but otherwise shows CMTV-like characteristics in regard to the global arrangement of its genome (Table 6, Fig. 5) may indicate that this virus represents an intermediate virus during ranavirus evolution. This isolate is a good example for the benefit of the newly developed PCR for rapid classification to the different variants of the genomic arrangement without elaborate full genome analyses.

By analyzing the relatively large set of ALRVs available based on whole genome nucleotide similarity and genome arrangements, it is evident that these are divided into three groups: EHNV-like, CMTV-like and FV3-like ranaviruses. The phylogenies however indicate that this split is very recent, more so among the FV3- and CMTV-like groups, which cannot be very confidently distinguished based on distance trees. As these two groups include pathogens of reptiles, amphibians, and fish, it is possible that this evolutionary split reflects geographic isolation rather than host-specific adaptations.

The capacity of interspecies and interclass transmission is an alarming feature of ranaviruses and may have contributed to their current worldwide emergence in a wide range of ectothermic vertebrates. Previous studies demonstrated that the spread of these pathogens to naïve geographic regions and species via commercial amphibian and fish trade (bait, pets, food industry) may be a possible mechanism for emergence [4, 48, 58]. This is the first study to compare a large number of ranaviruses detected in reptiles to isolates from other ectothermic vertebrates and provides three complete ranaviral genomes isolated from reptiles. These newly obtained reptilian ranavirus genomes, which are clearly distinct from each other and from FV3, provide valuable information comparing evolutionary traits and possible host determinants. Our results indicate that the role of reptiles in the epidemiology of ranaviral disease may be underestimated and that the trade with reptiles should also be considered as an important means of pathogen pollution. Future complete sequencing of more reptilian ranaviruses and comparison between large sets of isolates focusing on specific genes involved in virulence and host switching, as well as transmission studies are needed to understand the mechanisms involved in their evolution and emergence and will help to further study this ongoing unique phenomenon of viral adaptive radiation.

## Conclusion

This study compares a panel of ranaviruses detected in Europe in a wide range of captive and wild reptilian (n = 10) and amphibian (n = 4) hosts to each other and to previously studied isolates from ectothermic vertebrates based on seven genomic regions. Most of the viruses studied differed from one another based on partial sequences of the studied genes (MCP, DNApol, RNR-α and-β), but several closely related FV3-like isolates could only be distinguished based on concatenated sequences of at least three genes or by visualisation of the highly variable microsatellite region. The length of a potential virulence factor (vIF-2α) did not clearly correlate with the observed clinical signs in the infected animals, suggesting that another protein or host factors may contribute to the course of infection. The complete genomes from three reptilian ranaviruses were analyzed and specific genomic arrangement sites were studied to classify all viruses to one of the proposed ALRV groups. In the phylogenetic studies, the reptilian ranaviruses clustered often more closely to amphibian ranaviruses (FV3-like, TFV-like or CMTV-like) detected in the same geographic area of origin. These findings support the host-switch theory and stress the potential role of the animal trade with reptiles in the epidemiology of ranaviral disease.

## Supporting Information

S1 Table Ranavirus sequence percent identity values based on the partial MCP gene (1332nt). The twelve newly studied ranaviruses (CH8/96, ToRV1, ToRV2, GGRV, LMRV, JSpRV, ASRV, DGRV, ACRV, BPRV, NCRV, and PNTRV) are presented in comparison to selected previously studied ranavirus isolates (STIV, ZPRV1, ZPRV2, FV3, ATV, BIV, RGVl, TFV, ADRV, CMTV, EHNV, ESV). The upper diagonal shows the values for the nucleotide sequence identity, the amino acid identity values are provided in the lower diagonal. Highest identity values are highlighted bold.
CH8/96: Testudo hermanni ranavirus; ToRV1 and 2: tortoise ranavirus 1 and 2; STIV: soft-shelled turtle iridovirus; GGRV: German gecko ranavirus; LMRV: Lacerta monticola ranavirus; JSpRV: Japalura splendida ranavirus; ASRV: Anolis sagrei ranavirus; DGRV: Dopasia gracilis ranavirus; ACRV: Anolis carolinensis ranavirus; BPRV: blood python ranavirus; ZPRV1 and 2: Zuerich Pelophylax collection ranavirus 1 and 2; NCRV: Neurergus crocatus ranavirus; PNTRV: Portuguese newt and toad ranavirus; FV3: *Frog virus 3*; ATV: *Ambystoma tigrinum virus*; BIV: *Bohle iridovirus*; RGV: Rana grylio virus; TFV: tiger frog virus; ADRV: Andrias davidianus ranavirus; CMTV: common midwife toad virus; EHNV: *Epizootic haematopoietic necrosis virus*; ESV: European sheatfish virus; GenBank accession numbers are provided in Tables 1–3.
(DOC)

S2 Table Ranavirus sequence percent identity values based on the partial DNA polymerase gene (519nt). The twelve newly studied ranaviruses (CH8/96, ToRV1, ToRV2, GGRV, LMRV, JSpRV, ASRV, DGRV, ACRV, BPRV, NCRV, and PNTRV) are presented in comparison to selected previously studied ranavirus isolates (STIV, ZPRV1, ZPRV2, FV3, ATV, BIV, RGV, TFV, ADRV, CMTV, EHNV, ESV). The upper diagonal shows the values for the nucleotide sequence identity, the amino acid identity values are provided in the lower diagonal. Highest identity values are highlighted bold.
Full virus names are given in S1 Table; GenBank accession numbers are provided in Tables 1–3.
(DOC)

S3 Table Ranavirus sequence percent identity values based on the partial RNR-α gene (764 nt). The twelve newly studied ranaviruses (CH8/96, ToRV1, ToRV2, GGRV, LMRV, JSpRV, ASRV, DGRV, ACRV, BPRV, NCRV, and PNTRV) are presented in comparison to selected previously studied ranavirus isolates (STIV, ZPRV1, ZPRV2, FV3, ATV, BIV, RGV, TFV, ADRV, CMTV, EHNV, ESV). The upper diagonal shows the values for the nucleotide sequence identity, the amino acid identity values are provided in the lower diagonal. Highest identity values are highlighted bold.
Full virus names are given in S1 Table; GenBank accession numbers are provided in Tables 1–3.
(DOC)

S4 Table Ranavirus sequence percent identity values based on the partial RNR-β gene (608 nt). The twelve newly studied ranaviruses (CH8/96, ToRV1, ToRV2, GGRV, LMRV, JSpRV, ASRV, DGRV, ACRV, BPRV, NCRV, and PNTRV) are presented in comparison to selected previously studied ranavirus isolates (STIV, ZPRV1, ZPRV2, FV3, ATV, BIV, RGV, TFV, ADRV, CMTV, EHNV, ESV). The upper diagonal shows the values for the nucleotide sequence identity, the amino acid identity values are provided in the lower diagonal. Highest identity values are highlighted bold.
Full virus names are given in S1 Table; GenBank accession numbers are provided in Tables 1–3.
(DOC)

S5 Table Ranavirus sequence percent identity values based on the partial *v*IF-2α gene. The eleven newly studied ranaviruses with a complete *v*IF-2α gene (CH8/96, ToRV1, ToRV2, GGRV, ASRV, DGRV, BPRV, ZPRV1, ZPRV2, NCRV, and PNTRV) are presented in comparison to selected previously studied ranavirus isolates with a full-length *v*IF-2α gene (ATV, BIV, TFV, ADRV, CMTV, EHNV, ESV). The upper diagonal shows the values for the nucleotide sequence identity, the amino acid identity values are provided in the lower diagonal.
Full virus names are given in S1 Table; GenBank accession numbers used in this analysis are provided in Tables 1–3.
(DOC)

S6 Table Analyses of the new sequenced full-length genomes. Full virus names are given in S1 Table; GenBank accession numbers used in this analysis are provided in Tables 1 and 2.
(DOC)
