# Supplementary material for: Phylogeny and Differentiation of Reptilian and Amphibian Ranaviruses Detected in Europe
Source: PLoS One. 2015 Feb 23;10(2):e0118633. doi: 10.1371/journal.pone.0118633 (PMC4338083; doi:10.1371/journal.pone.0118633)
Supplement: S1 Table — The twelve newly studied ranaviruses (CH8/96, ToRV1, ToRV2, GGRV, LMRV, JSpRV, ASRV, DGRV, ACRV, BPRV, NCRV, and PNTRV) are presented in comparison to selected previously studied ranavirus isolates (STIV, ZPRV1, ZPRV2, FV3, ATV, BIV, RGVl, TFV, ADRV, CMTV, EHNV, ESV). The upper diagonal shows the values for the nucleotide sequence identity, the amino acid identity values are provided in the lower diagonal. Highest identity values are highlighted bold. CH8/96: Testudo hermanni ranavirus; ToRV1 and 2: tortoise ranavirus 1 and 2; STIV: soft-shelled turtle iridovirus; GGRV: German gecko ranavirus; LMRV: Lacerta monticola ranavirus; JSpRV: Japalura splendida ranavirus; ASRV: Anolis sagrei ranavirus; DGRV: Dopasia gracilis ranavirus; ACRV: Anolis carolinensis ranavirus; BPRV: blood python ranavirus; ZPRV1 and 2: Zuerich Pelophylax collection ranavirus 1 and 2; NCRV: Neurergus crocatus ranavirus; PNTRV: Portuguese newt and toad ranavirus; FV3: Frog virus 3; ATV: Ambystoma tigrinum virus; BIV: Bohle iridovirus; RGV: Rana grylio virus; TFV: tiger frog virus; ADRV: Andrias davidianus ranavirus; CMTV: common midwife toad virus; EHNV: Epizootic haematopoietic necrosis virus; ESV: European sheatfish virus; GenBank accession numbers are provided in Tables 1–3. (DOC) [file pone.0118633.s001.doc]

S1 Table: Ranavirus sequence percent identity values based on the partial MCP gene (1332nt). The twelve newly studied ranaviruses (CH8/96, ToRV1, ToRV2, GGRV, LMRV, JSpRV, ASRV, DGRV, ACRV, BPRV, NCRV and PNTRV) are presented in comparison to selected previously studied ranavirus isolates (STIV, ZPRV1, ZPRV2, FV3, ATV, BIV, RGV, TFV, ADRV, CMTV, EHNV, ESV). The upper diagonal shows the values for the nucleotide sequence identity, the amino acid identity values are provided in the lower diagonal.

|  | CH8/96 | ToRV1 | ToRV2 | STIV | GGRV | LMRV | JSpRV | ASRV | DGRV | ACRV | BPRV | ZPRV1 | ZPRV2 | NCRV | PNTRV | FV3 | ATV | BIV | RGV | TFV | ADRV | CMTV | EHNV | ESV |
| --- | --- | --- | --- | --- | --- | --- | --- | --- | --- | --- | --- | --- | --- | --- | --- | --- | --- | --- | --- | --- | --- | --- | --- | --- |
| CH8/96 |  | 98.1 | 98.1 | 98.4 | 98.4 | 98.3 | 98.3 | 98.1 | 98.0 | 98.3 | 97.5 | 99.6 | 99.7 | 99.8 | 99.4 | 98.2 | 97.4 | 98.4 | 98.4 | 98.3 | 99.7 | 99.4 | 98.9 | 98.2 |
| TRV1 | 98.7 |  | **100** | 98.1 | 98.1 | 98.0 | 98.0 | 97.8 | 97.7 | 98.9 | 97.2 | 98.0 | 98.1 | 98.0 | 97.9 | 97.9 | 96.3 | 98.1 | 98.1 | 98.0 | 98.1 | 98.2 | 97.7 | 97.0 |
| TRV2 | 98.4 | **100** |  | 98.1 | 98.1 | 98.0 | 98.0 | 97.8 | 97.7 | 98.9 | 97.2 | 98.0 | 98.1 | 98.0 | 97.9 | 97.9 | 96.3 | 98.1 | 98.1 | 98.0 | 98.1 | 98.2 | 97.7 | 97.0 |
| STIV | 98.1 | 99.3 | 99.3 |  | 98.7 | 99.7 | 99.7 | 99.3 | 98.2 | 99.7 | 97.7 | 98.2 | 98.3 | 98.2 | 98.1 | 99.6 | 96.3 | 98.8 | **100** | 98.5 | 98.3 | 98.6 | 97.8 | 97.2 |
| GGRV | 98.6 | 99.7 | 99.7 | 99.5 |  | 98.7 | 98.7 | 98.7 | 98.1 | 98.7 | 97.5 | 98.2 | 98.3 | 98.2 | 98.0 | 98.6 | 96.3 | 99.6 | 98.7 | 98.4 | 98.3 | 98.4 | 97.8 | 96.9 |
| LMRV | 97.9 | 99.0 | 99.0 | 99.7 | 99.3 |  | **100** | 99.5 | 98.1 | **100** | 97.6 | 98.1 | 98.2 | 98.1 | 98.1 | 99.9 | 96.2 | 98.7 | 99.7 | 98.4 | 98.2 | 98.5 | 97.7 | 97.2 |
| JSRV | 97.9 | 99.0 | 99.0 | 99.7 | 99.3 | **100** |  | 99.5 | 98.1 | **100** | 97.6 | 98.1 | 98.2 | 98.1 | 98.1 | 99.9 | 96.2 | 98.7 | 99.7 | 98.4 | 98.2 | 98.5 | 97.7 | 97.2 |
| ASRV | 97.9 | 99.0 | 99.0 | 99.3 | 99.3 | 99.5 | 99.5 |  | 98.0 | 99.5 | 97.5 | 98.0 | 98.1 | 98.0 | 97.9 | 99.4 | 96.2 | 98.7 | 99.3 | 98.3 | 98.1 | 98.4 | 97.5 | 96.9 |
| DGRV | 97.5 | 98.6 | 98.6 | 98.4 | 98.8 | 98.1 | 98.1 | 98.1 |  | 98.1 | 98.7 | 97.8 | 97.9 | 98.0 | 97.8 | 98.1 | 96.2 | 98.1 | 98.2 | 99.2 | 97.9 | 98.4 | 97.4 | 96.7 |
| ACRV | 97.9 | 99.0 | 99.0 | 99.7 | 99.3 | **100** | **100** | 99.5 | 98.1 |  | 97.6 | 98.1 | 98.2 | 98.1 | 98.1 | 99.9 | 96.2 | 98.7 | 99.7 | 98.4 | 98.2 | 98.5 | 97.7 | 97.2 |
| PBRV | 96.8 | 97.7 | 97.7 | 97.5 | 97.9 | 97.2 | 97.2 | 97.2 | 99.0 | 97.2 |  | 97.5 | 97.5 | 97.4 | 97.3 | 97.5 | 95.5 | 97.6 | 97.7 | 98.7 | 97.5 | 97.7 | 96.9 | 96.2 |
| ZPRV1 | **100** | 98.4 | 98.4 | 98.1 | 98.6 | 97.9 | 97.9 | 97.9 | 97.5 | 97.9 | 96.8 |  | 99.7 | 99.5 | 99.3 | 98.1 | 97.3 | 98.3 | 98.2 | 98.1 | 99.7 | 99.3 | 98.7 | 98.1 |
| ZPRV2 | **100** | 98.4 | 98.4 | 98.1 | 98.6 | 97.9 | 97.9 | 97.9 | 97.5 | 97.9 | 96.8 | **100** |  | 99.6 | 99.3 | 98.1 | 97.3 | 98.4 | 98.3 | 98.2 | 99.8 | 99.5 | 98.8 | 98.1 |
| NCRV | **100** | 98.4 | 98.4 | 98.1 | 98.6 | 97.9 | 97.9 | 97.9 | 97.5 | 97.9 | 96.8 | **100** | **100** |  | 99.3 | 98.1 | 97.4 | 98.3 | 98.2 | 98.1 | 99.6 | 99.4 | 98.7 | 98.1 |
| PARV | 99.5 | 98.4 | 98.4 | 97.7 | 98.1 | 97.5 | 97.5 | 97.5 | 97.0 | 97.5 | 96.3 | 99.5 | 99.5 | 99.5 |  | 98.0 | 97.2 | 98.1 | 98.1 | 98.1 | 99.3 | 99.2 | 98.7 | 98.1 |
| FV3 | 97.7 | 98.8 | 98.8 | 99.5 | 99.0 | 99.7 | 99.7 | 99.3 | 97.9 | 99.7 | 97.0 | 97.7 | 97.7 | 97.7 | 97.2 |  | 96.1 | 98.7 | 99.6 | 98.4 | 98.1 | 98.4 | 97.6 | 97.1 |
| ATV | 97.7 | 96.1 | 96.1 | 95.9 | 96.3 | 95.7 | 95.7 | 95.7 | 95.7 | 95.7 | 95.0 | 97.7 | 97.7 | 97.7 | 97.2 | 95.4 |  | 96.3 | 96.3 | 96.3 | 97.3 | 97.5 | 98.0 | 97.3 |
| BIV | 98.6 | 99.7 | 99.7 | 99.5 | **100** | 99.3 | 99.3 | 99.3 | 98.8 | 99.3 | 97.9 | 98.6 | 98.6 | 98.6 | 98.1 | 99.0 | 96.3 |  | 98.8 | 98.4 | 98.4 | 98.5 | 97.8 | 97.0 |
| RGV | 98.1 | 99.3 | 99.3 | **100** | 99.5 | 99.7 | 99.7 | 99.3 | 98.4 | 99.7 | 97.5 | 98.1 | 98.1 | 98.1 | 97.7 | 99.5 | 95.9 | 99.5 |  | 98.5 | 98.3 | 98.6 | 97.8 | 97.2 |
| TFV | 97.7 | 98.8 | 98.8 | 98.6 | 99.0 | 98.4 | 98.4 | 98.4 | 99.7 | 98.4 | 98.8 | 97.7 | 97.7 | 97.7 | 97.2 | 98.1 | 95.9 | 99.0 | 98.6 |  | 98.2 | 98.5 | 97.7 | 97.0 |
| ADRV | **100** | 98.4 | 98.4 | 98.1 | 98.6 | 97.9 | 97.9 | 97.9 | 97.5 | 97.9 | 96.8 | **100** | **100** | **100** | 99.5 | 97.7 | 97.7 | 98.6 | 98.1 | 97.7 |  | 99.3 | 98.8 | 98.1 |
| CMTV | 99.7 | 98.6 | 98.6 | 98.4 | 98.8 | 98.1 | 98.1 | 98.1 | 97.7 | 98.1 | 96.8 | 99.7 | 99.7 | 99.7 | 99.3 | 97.9 | 97.5 | 98.8 | 98.4 | 97.9 | 99.7 |  | 98.8 | 98.1 |
| EHNV | 98.8 | 97.2 | 97.2 | 97.0 | 97.5 | 96.8 | 96.8 | 96.8 | 96.3 | 96.8 | 95.7 | 98.8 | 98.8 | 98.8 | 98.4 | 96.6 | 97.9 | 97.5 | 97.0 | 96.6 | 98.8 | 98.6 |  | 98.8 |
| ESV | 97.5 | 95.9 | 95.9 | 96.1 | 96.1 | 95.9 | 95.9 | 95.4 | 95.2 | 95.9 | 94.8 | 97.5 | 97.5 | 97.5 | 97.0 | 95.7 | 96.8 | 96.1 | 96.1 | 95.4 | 97.5 | 97.2 | 97.7 |  |

CH8/96: Testudo hermanni ranavirus; ToRV1 and 2: tortoise ranavirus 1 and 2; STIV: soft-shelled turtle iridovirus; GGRV: German gecko ranavirus; LMRV: Lacerta monticola ranavirus; JSpRV: Japalura splendida ranavirus; ASRV: Anolis sagrei ranavirus; DGRV: Dopasia gracilis ranavirus; ACRV: Anolis carolinensis ranavirus; PBRV: blood python ranavirus; ZPRV1 and 2: Zuerich Pelophylax collection ranavirus 1 and 2; NCRV: Neurergus crocatus ranavirus; PNTRV: Portuguese newt and toad ranavirus; FV3: *Frog virus 3*; ATV: *Ambystoma tigrinum virus*; BIV: *Bohle iridovirus*; RGV: Rana grylio virus; TFV: tiger frog virus; ADRV: Andrias davidianus ranavirus; CMTV: common midwife toad virus; EHNV: *Epizootic haematopoietic necrosis virus*; GenBank accession numbers are provided in legend to Figure 3 and in Table 1.
