# Supplementary material for: Phylogeny and Differentiation of Reptilian and Amphibian Ranaviruses Detected in Europe
Source: PLoS One. 2015 Feb 23;10(2):e0118633. doi: 10.1371/journal.pone.0118633 (PMC4338083; doi:10.1371/journal.pone.0118633)
Supplement: S2 Table — The twelve newly studied ranaviruses (CH8/96, ToRV1, ToRV2, GGRV, LMRV, JSpRV, ASRV, DGRV, ACRV, BPRV, NCRV, and PNTRV) are presented in comparison to selected previously studied ranavirus isolates (STIV, ZPRV1, ZPRV2, FV3, ATV, BIV, RGV, TFV, ADRV, CMTV, EHNV, ESV). The upper diagonal shows the values for the nucleotide sequence identity, the amino acid identity values are provided in the lower diagonal. Highest identity values are highlighted bold. Full virus names are given in S1 Table; GenBank accession numbers are provided in Tables 1–3. (DOC) [file pone.0118633.s002.doc]

S2 Table: Ranavirus sequence percent identity values based on the partial DNApolymerase gene (519nt). The twelve newly studied ranaviruses (CH8/96, ToRV1, ToRV2, GGRV, LMRV, JSRV, ASRV, DGRV, ACRV, BPRV, NCRV and PNTRV) are presented in comparison to selected previously studied ranavirus isolates (STIV, ZPRV1, ZPRV2, FV3, ATV, BIV, RGV, TFV, ADRV, CMTV, EHNV, ESV). The upper diagonal shows the values for the nucleotide sequence identity, the amino acid identity values are provided in the lower diagonal.

|  | CH8/96 | ToRV1 | ToRV2 | STIV | GGRV | LMRV | JSpRV | ASRV | DGRV | ACRV | BPRV | ZPRV1 | ZPRV2 | NCRV | PNTRV | FV3 | ATV | BIV | RGV | TFV | ADRV | CMTV | EHNV | ESV |
| --- | --- | --- | --- | --- | --- | --- | --- | --- | --- | --- | --- | --- | --- | --- | --- | --- | --- | --- | --- | --- | --- | --- | --- | --- |
| CH8/96 |  | 98.4 | 98.6 | 99.0 | 98.0 | 99.0 | 90.0 | 99.2 | 98.6 | 99.0 | 98.2 | 99.0 | 99.2 | 98.2 | 99.2 | 99.0 | 98.2 | 98.6 | 98.6 | 98.6 | 99.4 | 98.4 | 98.4 | 98.8 |
| TRV1 | 98.2 |  | 99.8 | 98.6 | 97.6 | 98.6 | 98.6 | 98.8 | 98.2 | 98.6 | 97.8 | 98.6 | 98.4 | 97.4 | 98.4 | 98.6 | 97.8 | 98.2 | 98.2 | 98.2 | 98.2 | 98.2 | 98.0 | 98.4 |
| TRV2 | 98.2 | **100** |  | 98.8 | 97.8 | 98.8 | 98.8 | 99.0 | 98.4 | 98.8 | 98.0 | 98.4 | 98.6 | 97.6 | 98.6 | 98.8 | 98.0 | 98.4 | 98.4 | 98.4 | 98.4 | 98.2 | 98.2 | 98.6 |
| STIV | 99.4 | 98.8 | 98.8 |  | 98.2 | 99.6 | 99.6 | 99.4 | 98.8 | 99.6 | 98.4 | 98.8 | 99.0 | 98.0 | 99.0 | 99.6 | 98.4 | 98.8 | 99.6 | 98.8 | 98.8 | 98.6 | 98.6 | 99.0 |
| GGRV | 98.2 | 97.6 | 97.6 | 98.8 |  | 98.2 | 98.2 | 98.4 | 98.2 | 98.2 | 97.8 | 97.8 | 98.0 | 97.1 | 98.0 | 98.2 | 97.8 | 99.4 | 97.8 | 98.2 | 98.0 | 97.6 | 98.0 | 98.4 |
| LMRV | 98.8 | 98.2 | 98.2 | 99.4 | 98.2 |  | 99.6 | 99.8 | 99.0 | 99.6 | 98.4 | 98.8 | 99.0 | 98.0 | 99.0 | **100** | 98.4 | 98.8 | 99.2 | 98.8 | 98.8 | 98.6 | 98.6 | 99.0 |
| JSRV | 98.8 | 98.2 | 98.2 | 99.4 | 98.2 | 98.8 |  | 99.4 | 98.8 | **100** | 98.8 | 98.8 | 99.0 | 98.0 | 99.0 | 99.6 | 98.4 | 98.8 | 99.2 | 98.8 | 98.8 | 98.6 | 98.6 | 99.0 |
| ASRV | 98.8 | 98.2 | 98.2 | 99.4 | 98.2 | **100** | 98.8 |  | 99.2 | 99.4 | 98.6 | 99.0 | 99.2 | 98.2 | 99.2 | 99.8 | 98.6 | 99.0 | 99.0 | 99.0 | 99.0 | 98.8 | 98.8 | 99.2 |
| DGRV | 98.2 | 97.6 | 97.6 | 98.8 | 98.8 | 98.2 | 98.2 | 98.2 |  | 98.8 | 98.8 | 98.4 | 98.6 | 97.6 | 98.6 | 99.0 | 98.4 | 98.4 | 98.4 | 99.2 | 98.6 | 98.2 | 98.6 | 99.0 |
| ACRV | 98.8 | 98.2 | 98.2 | 99.4 | 98.2 | 98.8 | **100** | 98.8 | 98.2 |  | 98.8 | 98.8 | 99.0 | 98.0 | 99.0 | 99.6 | 98.4 | 98.8 | 99.2 | 98.8 | 98.8 | 98.6 | 98.6 | 99.0 |
| PBRV | 97.1 | 96.5 | 96.5 | 97.6 | 97.6 | 97.1 | 98.2 | 97.1 | 97.6 | 98.2 |  | 98.0 | 98.2 | 97.3 | 98.2 | 98.4 | 98.0 | 98.0 | 98.0 | 98.8 | 98.2 | 97.8 | 98.2 | 98.6 |
| ZPRV1 | 99.4 | 97.6 | 97.6 | 98.8 | 97.6 | 98.2 | 98.2 | 98.2 | 97.6 | 98.2 | 96.5 |  | 99.8 | 98.0 | 99.4 | 98.8 | 98.0 | 98.4 | 98.4 | 98.4 | 98.8 | 98.2 | 98.2 | 98.6 |
| ZPRV2 | 99.4 | 97.6 | 97.6 | 98.8 | 97.6 | 98.2 | 98.2 | 98.2 | 97.6 | 98.2 | 96.5 | **100** |  | 98.2 | 99.6 | 99.0 | 98.2 | 98.6 | 98.6 | 98.6 | 99.0 | 98.4 | 98.4 | 98.9 |
| NCRV | 97.1 | 95.3 | 95.3 | 96.5 | 95.3 | 95.9 | 95.9 | 95.9 | 95.3 | 95.9 | 94.2 | 96.5 | 96.5 |  | 98.2 | 98.0 | 98.0 | 97.6 | 97.6 | 97.6 | 98.0 | 97.4 | 97.8 | 98.2 |
| PARV | 99.4 | 97.6 | 97.6 | 98.8 | 97.6 | 98.2 | 98.2 | 98.2 | 97.6 | 98.2 | 96.5 | **100** | **100** | 96.5 |  | 99.0 | 98.2 | 98.6 | 98.6 | 98.6 | 99.0 | 98.4 | 98.4 | 98.8 |
| FV3 | 98.8 | 98.2 | 98.2 | 99.4 | 98.2 | **100** | 98.8 | **100** | 98.2 | 98.8 | 97.1 | 98.2 | 98.2 | 95.9 | 98.2 |  | 98.4 | 98.8 | 99.2 | 98.8 | 98.8 | 98.6 | 98.6 | 99.0 |
| ATV | 97.6 | 97.1 | 97.1 | 98.2 | 98.2 | 97.6 | 97.6 | 97.6 | 98.2 | 97.6 | 97.6 | 97.1 | 97.1 | 95.9 | 97.1 | 97.6 |  | 98.0 | 98.0 | 98.4 | 98.2 | 97.8 | 99.0 | 99.4 |
| BIV | 98.8 | 98.2 | 98.2 | 99.4 | 99.4 | 98.8 | 98.8 | 98.8 | 98.2 | 98.8 | 97.1 | 98.2 | 98.2 | 95.9 | 98.2 | 98.8 | 97.6 |  | 98.4 | 98.4 | 98.4 | 98.2 | 98.2 | 98.6 |
| RGV | 98.2 | 97.6 | 97.6 | 98.8 | 97.6 | 98.2 | 98.2 | 98.2 | 97.6 | 98.2 | 96.5 | 97.6 | 97.6 | 95.3 | 97.6 | 98.2 | 97.1 | 98.2 |  | 98.4 | 98.4 | 98.2 | 98.2 | 98.6 |
| TFV | 97.6 | 97.1 | 97.1 | 98.2 | 98.2 | 97.6 | 97.6 | 97.6 | 98.2 | 97.6 | 97.1 | 97.1 | 97.1 | 94.7 | 97.1 | 97.6 | 97.6 | 97.6 | 97.1 |  | 98.6 | 98.2 | 98.6 | 99.0 |
| ADRV | 99.4 | 97.6 | 97.6 | 98.8 | 98.2 | 98.2 | 98.2 | 98.2 | 98.2 | 98.2 | 97.1 | 98.8 | 98.8 | 96.5 | 98.8 | 98.2 | 97.6 | 98.2 | 97.6 | 97.6 |  | 98.2 | 98.4 | 98.8 |
| CMTV | 97.6 | 97.1 | 97.1 | 98.2 | 97.1 | 97.6 | 97.6 | 97.6 | 97.1 | 97.6 | 95.9 | 97.1 | 97.1 | 94.7 | 97.1 | 97.6 | 96.5 | 97.6 | 97.1 | 96.5 | 97.1 |  | 98.0 | 98.4 |
| EHNV | 98.2 | 97.6 | 97.6 | 98.8 | 98.8 | 98.2 | 98.2 | 98.2 | 98.8 | 98.2 | 97.6 | 97.6 | 97.6 | 95.3 | 97.6 | 98.2 | 98.2 | 98.2 | 97.6 | 98.2 | 98.2 | 97.1 |  | 99.6 |
| ESV | 98.8 | 98.2 | 98.2 | 99.4 | 99.4 | 98.8 | 98.8 | 98.8 | 99.4 | 98.8 | 98.2 | 98.2 | 98.2 | 95.9 | 98.2 | 98.8 | 98.8 | 98.8 | 98.2 | 98.8 | 98.8 | 97.6 | 99.4 |  |

Full virus names are given in S1 Table; GenBank accession numbers are provided in legend to Figure 3 and in Table 1.
