# Supplementary material for: Phylogeny and Differentiation of Reptilian and Amphibian Ranaviruses Detected in Europe
Source: PLoS One. 2015 Feb 23;10(2):e0118633. doi: 10.1371/journal.pone.0118633 (PMC4338083; doi:10.1371/journal.pone.0118633)
Supplement: S5 Table — The eleven newly studied ranaviruses with a complete vIF-2α gene (CH8/96, ToRV1, ToRV2, GGRV, ASRV, DGRV, BPRV, ZPRV1, ZPRV2, NCRV, and PNTRV) are presented in comparison to selected previously studied ranavirus isolates with a full-length vIF-2α gene (ATV, BIV, TFV, ADRV, CMTV, EHNV, ESV). The upper diagonal shows the values for the nucleotide sequence identity, the amino acid identity values are provided in the lower diagonal. Full virus names are given in S1 Table; GenBank accession numbers used in this analysis are provided in Tables 1–3. (DOC) [file pone.0118633.s005.doc]

S5 Table: Ranavirus sequence percent identity values based on the partial *v*IF-2α gene gene. The eleven newly studied ranaviruses with a complete *v*IF-2α gene (CH8/96, ToRV1, ToRV2, GGRV, ASRV, DGRV, BPRV, ZPRV1, ZPRV2, NCRV and PNTRV) are presented in comparison to selected previously studied ranavirus isolates with a full-length *v*IF-2α gene (ATV, BIV, TFV, ADRV, CMTV, EHNV, ESV). The upper diagonal shows the values for the nucleotide sequence identity, the amino acid identity values are provided in the lower diagonal.

|  | CH8/96 | ToRV1 | ToRV2 | GGRV | ASRV | DGRV | BPRV | ZPRV1 | ZPRV2 | NCRV | PNTRV | ATV | BIV | TFV | ADRV | CMTV | EHNV | ESV |
| --- | --- | --- | --- | --- | --- | --- | --- | --- | --- | --- | --- | --- | --- | --- | --- | --- | --- | --- |
| CH8/96 |  | 95.0 | 95.0 | 97.4 | 98.4 | 96.6 | 96.4 | 98.8 | 99.1 | 98.1 | 98.0 | 95.7 | 97.6 | 97.0 | 99.2 | 98.3 | 97.4 | 95,7 |
| TRV1 | 93.4 |  | **100** | 94.6 | 94.2 | 93.7 | 93.3 | 94.7 | 94.8 | 94.1 | 94.2 | 93.2 | 94.8 | 93.7 | 94.9 | 94.7 | 94.2 | 92.6 |
| TRV2 | 93.4 | **100** |  | 94.6 | 94.2 | 93.7 | 93.3 | 94.7 | 94.8 | 94.1 | 94.2 | 93.2 | 94.8 | 93.7 | 94.9 | 94.7 | 94.2 | 92.6 |
| GGRV | 95.0 | 91.5 | 91.5 |  | 96.8 | 95.8 | 95.8 | 97.3 | 97.1 | 96.5 | 96.6 | 94.8 | 98.7 | 96.3 | 97.3 | 97.0 | 96.2 | 94.6 |
| ASRV | 97.1 | 91.8 | 91.8 | 94.0 |  | 95.8 | 95.7 | 98.1 | 98.8 | 97.6 | 97.3 | 95.0 | 97.0 | 96.2 | 98.9 | 97.6 | 96.6 | 95.0 |
| DGRV | 93.1 | 90.3 | 90.3 | 91.9 | 91.5 |  | 96.5 | 96.2 | 96.4 | 95.7 | 95.6 | 94.2 | 96.0 | 96.8 | 96.4 | 95.9 | 95.4 | 93.9 |
| PBRV | 93.1 | 89.9 | 89.9 | 91.8 | 91.5 | 93.7 |  | 96.1 | 96.3 | 95.6 | 95.6 | 94.0 | 95.9 | 96.8 | 96.5 | 95.8 | 95.3 | 93.6 |
| ZPRV1 | 97.4 | 92.1 | 92.1 | 94.3 | 95.9 | 92.2 | 92.1 |  | 98.6 | 97.8 | 97.7 | 95.6 | 97.5 | 96.6 | 98.7 | 98.0 | 97.0 | 95.4 |
| ZPRV2 | 98.7 | 92.7 | 92.7 | 94.3 | 97.8 | 92.8 | 92.4 | 96.8 |  | 97.9 | 97.8 | 95.5 | 97.4 | 96.8 | 99.6 | 98.1 | 97.1 | 95.5 |
| NCRV | 96.5 | 92.1 | 92.1 | 93.7 | 95.6 | 91.5 | 91.5 | 95.2 | 95.9 |  | 97.1 | 95.3 | 96.7 | 96.1 | 98.0 | 97.5 | 96.5 | 94.9 |
| PARV | 96.2 | 91.5 | 91.5 | 93.1 | 94.6 | 91.2 | 91.2 | 94.9 | 95.6 | 94.6 |  | 94.8 | 96.6 | 96.2 | 97.9 | 97.8 | 96.4 | 94.9 |
| ATV | 93.7 | 90.5 | 90.5 | 91.8 | 92.1 | 90.3 | 90.2 | 93.1 | 93.1 | 92.7 | 92.4 |  | 94.8 | 94.2 | 95.6 | 95.1 | 95.6 | 94.0 |
| BIV | 95.6 | 92.7 | 92.7 | 98.1 | 94.6 | 92.5 | 92.4 | 94.9 | 94.9 | 94.3 | 93.7 | 91.8 |  | 96.5 | 97.5 | 97.3 | 96.4 | 94.8 |
| TFV | 93.1 | 89.9 | 89.9 | 91.8 | 91.5 | 93.1 | 94.0 | 92.1 | 92.7 | 91.5 | 91.2 | 89.6 | 92.4 |  | 97.0 | 96.7 | 95.9 | 94.3 |
| ADRV | 98.7 | 92.7 | 92.7 | 94.3 | 97.8 | 92.5 | 93.1 | 96.8 | 99.3 | 95.9 | 95.6 | 93.1 | 94.9 | 93.1 |  | 98.2 | 97.3 | 95.6 |
| CMTV | 97.5 | 93.1 | 93.1 | 94.6 | 95.9 | 92.5 | 92.7 | 96.2 | 96.8 | 95.9 | 96.8 | 93.1 | 95.2 | 93.7 | 96.8 |  | 96.7 | 95.3 |
| EHNV | 95.6 | 91.5 | 91.5 | 93.1 | 94.0 | 91.2 | 91.2 | 94.3 | 94.9 | 94.0 | 93.7 | 92.7 | 93.7 | 90.9 | 94.9 | 94.9 |  | 96.0 |
| ESV | 94.1 | 90.1 | 90.1 | 91.6 | 92.5 | 90.1 | 89.8 | 92.9 | 93.5 | 92.5 | 92.2 | 91.6 | 92.2 | 89.5 | 93.5 | 93.5 | 94.1 |  |

Full virus names are given in S1 Table; GenBank accession numbers used in this analysis: ATV (AY150217), BIV (EF408913), TFV (AF389451), ADRV (KC865735), CMTV (JQ231222), EHNV (FJ433873), ESV (JQ724856).
