# Supplementary material for: Phylogeny and Differentiation of Reptilian and Amphibian Ranaviruses Detected in Europe
Source: PLoS One. 2015 Feb 23;10(2):e0118633. doi: 10.1371/journal.pone.0118633 (PMC4338083; doi:10.1371/journal.pone.0118633)
Supplement: S6 Table — Full virus names are given in S1 Table; GenBank accession numbers used in this analysis are provided in Tables 1 and 2. (DOC) [file pone.0118633.s006.doc]

S6 Table: Analyses of the new sequenced full-length genomes.

| Isolate | Number of bases | Number of annotated genes | Density | Average lenght | Coding percentage | Overall GC percentage |
| --- | --- | --- | --- | --- | --- | --- |
| GGRV | 103681 | 73 | 0.704 genes/kb (1420 bases/ gene) | 1092 | 76.9 | 55.06 |
| ToRV1 | 103876 | 76 | 0.731 genes/kb (1366 bases/gene) | 1084 | 79.3 | 55.22 |
| CH8/96 | 105811 | 75 | 0.708 genes/kb (1410 bases/gene) | 1082 | 76.7 | 55.37 |

Full virus names are given in S1 Table; GenBank accession numbers used in this analysis: GGRV (KP266742), ToRV1 (KP266743), CH8/96 (KP266741).
